# NMR Metabolomics Defining Genetic Variation in Pea Seed Metabolites

**DOI:** 10.3389/fpls.2018.01022

**Published:** 2018-07-17

**Authors:** Noel Ellis, Chie Hattori, Jitender Cheema, James Donarski, Adrian Charlton, Michael Dickinson, Giampaolo Venditti, Péter Kaló, Zoltán Szabó, György B. Kiss, Claire Domoney

**Affiliations:** ^1^John Innes Centre, Norwich, United Kingdom; ^2^IBERS, Aberystwyth University, Aberystwyth, United Kingdom; ^3^Faculty of Science, School of Biological Sciences, University of Auckland, Auckland, New Zealand; ^4^Fera Science Ltd., York, United Kingdom; ^5^National Agricultural Research and Innovation Centre, Agricultural Biotechnology Institute, Gödöllő, Hungary; ^6^AMBIS Biotechnology Ltd., Budapest, Hungary

**Keywords:** genetic map, genetic variation, pea, seed, metabolite, nuclear magnetic resonance

## Abstract

Nuclear magnetic resonance (NMR) spectroscopy profiling was used to provide an unbiased assessment of changes to the metabolite composition of seeds and to define genetic variation for a range of pea seed metabolites. Mature seeds from recombinant inbred lines, derived from three mapping populations for which there is substantial genetic marker linkage information, were grown in two environments/years and analyzed by non-targeted NMR. Adaptive binning of the NMR metabolite data, followed by analysis of quantitative variation among lines for individual bins, identified the main genomic regions determining this metabolic variability and the variability for selected compounds was investigated. Analysis by *t*-tests identified a set of bins with highly significant associations to genetic map regions, based on probability (*p*) values that were appreciably lower than those determined for randomized data. The correlation between bins showing high mean absolute deviation and those showing low *p*-values for marker association provided an indication of the extent to which the genetics of bin variation might be explained by one or a few loci. Variation in compounds related to aromatic amino acids, branched-chain amino acids, sucrose-derived metabolites, secondary metabolites and some unidentified compounds was associated with one or more genetic loci. The combined analysis shows that there are multiple loci throughout the genome that together impact on the abundance of many compounds through a network of interactions, where individual loci may affect more than one compound and *vice versa*. This work therefore provides a framework for the genetic analysis of the seed metabolome, and the use of genetic marker data in the breeding and selection of seeds for specific seed quality traits and compounds that have high commercial value.

## Introduction

Metabolite profiling, based on chemical fingerprints provided by nuclear magnetic resonance (NMR) spectroscopy, provides an approach for the unbiased assessment of changes in the content of small molecules in response to genetic and/or environmental factors. Such profiles provide a useful and rapid method for assessing the changes that occur in the metabolome as a consequence of plant genotype and/or the interaction between genotype and environment (Messerli et al., 2007). The use of NMR spectroscopy for holistic studies of plant metabolism predates the term “metabolomics” (Fiehn et al., 2000) by some years (Moore et al., 1983; Belton and Ratcliffe, 1985; Ratcliffe, 1987; Fan, 1996).

NMR spectroscopy provides a method of choice to facilitate the efficient analysis of the large number of samples that is necessary to deal with the expected intrinsic variability of plant, or equivalent, biological materials particularly where these need to be grown or cultured under field or similar “near-natural” conditions. Such has been the case for the study of “substantial equivalence” in genetically modified plants, where NMR has been used in the analysis of field samples of wheat (Baker et al., 2006). Higher amounts of maltose and/or sucrose and differences in free amino acids were apparent in a transgenic line, and these observations were followed by more detailed studies of the amino acid composition using gas chromatography-mass spectrometry (GC-MS). NMR has also been employed to evaluate the effects of genetic modification and assess the effect of drought-stress on the *Pisum sativum* L. (pea) leaf metabolome (Charlton et al., 2004, 2008). Significant changes in resonances under drought-stress conditions were attributed to a range of compounds, both primary and secondary metabolites, including proline, valine, threonine, homoserine, myoinositol, γ -aminobutyrate (GABA) and trigonelline (nicotinic acid betaine). Some of these changes translated to alterations in the seed metabolome in the same experiments (unpublished data).

It has been shown, using GC/MS analyses of Arabidopsis developing seeds, that the seed desiccation period is associated with a major increase in the levels of free metabolites; these include the nitrogen-rich amino acids (asparagine, lysine, and arginine), the aromatic amino acids (tryptophan, phenylalanine, tyrosine), serine, alanine, the non-proteinogenic amino acid GABA, TCA-cycle intermediates, fumarate and succinate, and the levels of sucrose, galactose, arabinose, trehalose, sorbitol, galactitol, gluconate-6-phosphate and glycerate (Fait et al., 2006). Few studies have been carried out to investigate the effects of genetic variation on the metabolite composition of seeds. For the seeds of many crops, quality traits may be defined in terms of the synthesis of a number of key metabolites, for example the concentration of 2-acetyl-1-pyrroline (2AP) in rice linked to fragrance quality (Shi et al., 2008). An alternative to the expensive and time-consuming GC/MS method for assaying 2AP content in breeding programmes is offered by the demonstration that the metabolite is controlled by a gene, betaine aldehyde dehydrogenase, for which allelic variation has been described (Shi et al., 2008).

In pea, the molecular basis for many seed quality traits is largely unknown. An exception to this is the understanding of the control of sucrose content at a gross level, where naturally occurring mutants with defects in starch biosynthesis have elevated sucrose contents in their seeds. Mutations at two genetic loci (*r* and *rb*) have been exploited in the development of some of the varied food uses of pea seeds (Wang et al., 1998). Studies have shown the many pleiotropic effects that mutations at *r* and *rb* exert on seed metabolism overall; these include changes to nitrogen/protein accumulation, water content and seed shape when compared with wild-type lines (Perez et al., 1993; Casey et al., 1998; Lyall et al., 2003). These alterations to seed composition can be mimicked to similar or greater extents in mutants induced either through chemical mutagenesis or transgenesis, where additional genes of starch biosynthesis have been targeted (Wang and Hedley, 1991; Wang et al., 1998; Weigelt et al., 2009). While the *r* and *rb* loci are determined by mutations in a starch-branching enzyme and the large subunit of ADP-glucose pyrophosphorylase (AGPase), respectively, (Bhattacharyya et al., 1990; Hylton and Smith, 1992), transgenic lines of pea expressing RNAi constructs targeting the small subunit(s) of AGPase have shown a very similar phenotype, when compared with wild-type lines (Weigelt et al., 2009).

In many legume species, oligosaccharides derived from galactinol and sucrose are synthesized in seeds. In pea, these comprise the raffinose oligosaccharide (RFO) group of compounds, which include stachyose and verbascose in addition to raffinose. Quantitative and qualitative variation for these compounds has been described for pea, lentil and *Medicago* (Frias et al., 1994, 1999; Karner et al., 2004; Vandecasteele et al., 2011). Although RFOs are generally regarded as anti-nutrients in seeds, research in Medicago suggests that these compounds are related to seed vigor (Vandecasteele et al., 2011), while additional studies highlight their role in plant stress responses (Nakabayashi and Kazuki, 2015).

The aim of this study was to determine the extent to which variation in the metabolome of mature seeds was under genetic control and to investigate the main types of compounds involved in such regulation. This information could be used further to identify genotypes that are enriched in particular seed components, some of which may be associated positively or negatively with quality and/or health-promoting traits. Given the knowledge of the impact of the allelic state at the *r* and *rb* loci (above) and the variation that exists within these genotypes with respect to seed maturation, we sought to assess the extent of metabolome variation within *r* and *rb* genotypes of pea. In this paper, we define a metabolite phenotype for seeds from genetically marked recombinant inbred *r* and *rb* mutant lines. We describe variation within the metabolome of mature seeds from the recombinant inbred lines, for which we provide substantial genetic marker information and a framework for the analysis of metabolite data in relation to genetic loci and markers. Furthermore, for some of the identified metabolites, candidate genes have been identified for the control of metabolite content.

## Materials and methods

### Plant materials

A selection of recombinant inbred lines (RILs) from three mapping populations (JI 281 × JI 399, 32 lines; JI 15 × JI 399, 38 lines; JI 15 × JI 1194, 26 lines) and their parent lines (all available from the JIC *Pisum* germplasm collection; https://www.seedstor.ac.uk/search-browseaccessions.php?idCollection=6) were grown in microplots (1 m^2^) at two locations, John Innes Centre, Norwich (JIC) and at the Processors & Growers Research Organization, Peterborough (PGRO), over two consecutive seasons (Year 1, 2011 and Year 2, 2012). The lines comprise 100 variant vining seed genotypes (either *r* or *rb* mutants), derived from crosses that have integrated genetic maps and are densely populated with genetic markers (Supplementary File S1). The JI 1194 parent is *r*, JI 399 is *rb*, and JI 281 and JI 15 are wild type for both *r* and *rb*.

Seeds were treated with Wakil seed treatment and sown directly into plots in bird-proof cages in the spring (March). Plants were irrigated and sprayed for protection against aphids as necessary. Mature (senesced) plants and their seeds were harvested together in July. Seeds were threshed and hand-picked to remove any foreign objects, while phenotype checks ensured the identity, integrity and purity of the genetic stock. From these, seed aliquots (approximately 6g) were prepared for NMR metabolite analysis.

### NMR analysis

The NMR profiles of Year 1 and Year 2 samples (mature seeds from *r* and *rb* RILs grown at two sites) were analyzed by ^1^H high resolution NMR spectroscopy. Dried pea samples were ground into a fine powder and extracted with 1:1 methanol: water (150 mg per 1.5 mL). Samples were vortexed for 30 min before centrifugation (20,817g for 10 min). Methanol was removed from 900 μL of every supernatant by passing a stream of nitrogen over the sample for approximately 1 h. Samples were lyophilized overnight and then re-constituted in 700 μL NMR sample buffer (250 mM sodium phosphate, pH 7.0; 0.5 mM trimethylsilyl propanoic acid, TSP, dissolved in D_2_O), centrifuged (20,917g for 10 min) and 540 μL transferred to a labeled NMR tube. Sodium azide (60 μL aliquot of 10 mM, dissolved in D_2_O) was added to every sample to prevent microbial growth before NMR analysis. Extracts were also produced from the seed material using deuterated chloroform to ensure that metabolites which were not soluble in deuterated phosphate buffer solution were analyzed.

All spectra were acquired using an 11.7 T Bruker 500 MHz NMR spectrometer equipped with a 5 mm TCI cryoprobe. Acquisition and processing of the raw data were performed by using Topspin 2.13 patch level 6 (Bruker, Germany). NMR parameters and the magnetic field homogeneity were optimized using a control pea seed extract. The magnetic field was locked on the deuterium signal of the D_2_O and the homogeneity was optimized. The free induction decay (FID) was recorded using a 30° ^1^H flip angle determined from a 90° pulse length of 11.25 μs. A relaxation delay of 3 s was inserted into the pulse sequence to ensure that quantitative data were acquired. Repetitions (256) of 65,536 complex points were collected over a spectral width of 7002.8 Hz, with the center of the spectrum at 500.1323546 MHz. The NMR probe head was maintained at a temperature of 300 K and the sample remained static during data collection. These parameters resulted in a total experiment time of approximately 45 min per sample.

### NMR data processing

The data were Fourier transformed and an interactive phase correction applied to the spectrum. A baseline correction was applied and the spectrum referenced to the TSP peak at 0 ppm, the area of which was set to unity for all processed spectra using FELIX software (Accelrys, San Diego, CA, USA). Spectral binning of the resonances was performed using bespoke software, Metabolab, a graphical user interface developed using the Matlab platform. Adaptive binning was applied to the data by using the undecimated wavelet transform at a predefined level to reduce the number of variables and limit the effect of the variation of chemical shifts (Davis et al., 2007). Using this approach on the data acquired for different experiments (2 years) resulted in a difference in the total bin number determined for the two data sets. However, the bin identities could be compared, based on their defined limits.

### NMR compound identification

The identification of metabolites was performed by comparing resonances in the bins with the resonances of spectral data available either from a list of standards present in an internal database or from literature. As a literature source, the following on-line NMR databases were used:

1. Madison Metabolomic Database: http://mmcd.nmrfam.wisc.edu
2. Human Metabolomics Database: http://www.hmdb.ca
3. Database of organic compounds: http://sdbs.db.aist.go.jp/

To assign the binned data, the profiles of all acquired spectra were superimposed to determine the range of chemical shifts of all resonances included in the binned area. Following identification of chemical shift values, the listed databases were interrogated and a list of the most likely candidate metabolites was formed. The spectrum of the candidate metabolite was compared with the spectra acquired from the samples either directly, by using the spectra of the compounds in the internal database, or indirectly, using the on-line NMR databases (1–3 above). One-dimensional ^1^H and two-dimensional homonuclear and heteronuclear correlation NMR experiments (^1^H –^1^H TOCSY and ^13^C – ^1^H HSQC) were also used to aid the assignment. The acquisition parameters for the TOCSY and HSQC experiments are given in Supplementary Table 1. A set of resonances was attributed to aglycone derivatives of anthocyanins, based on the study of Kirby et al. (2013).

### Normalization of NMR bin data and determination of the mean of absolute deviation (MAD)

The analysis of variation within any one bin across RILs was carried out following normalization of the bin values to a mean of 100 and standard deviation of 1, which resulted in all values being positive. Binned NMR data were normalized according to the formula below to facilitate further data processing.

A'_L_ is the normalized bin area for the line L calculated as follows:
a_L_ = NMR peak area
mu_a_ = mean of the peak area for the RILs and the parents of the mapping population
SD_a_ = standard deviation of the peak area for the RILs and the parents of the mapping population
A'_L_ = 100 + ((a_L_ - mu_a_)/ SD_a_)
As a result, each bin has a mean of 100 and a standard deviation of 1.

Due to the number of data points to be analyzed (968 NMR bins for year 1, 990 NMR bins for year 2), an initial prioritization of bins for mapping analysis was achieved, using Mean of Absolute Deviation from the mean (MAD) values as a measure of the variation within any given bin. MAD values provide a measure of the absolute deviations of a set of data about the data's mean, that is, it is the average distance of the data set from its mean. Although high MAD values indicate bins with high variation across the population, this variation in phenotype does not necessarily indicate genetic variation. However, analysis of bins which have higher MAD values increases the possibility of detecting those bins for which quantitative trait loci (QTL) could be mapped. Due to the normalization carried out (as above), MAD values for the normalized data ranged between 0 and 1. Heat maps were generated to visualize MAD value distributions along the NMR spectrum and to compare relative MAD values among datasets (Supplementary Figure 1).

Linkage map analysis (see below) was performed for bins of interest, whereby quantitative variation within NMR bins was shown to be associated with genetic loci, if the two groups of lines carrying one or the other parent marker at that locus showed significant difference in NMR signal strength. Analysis by *t*-tests identified a set of bins with highly significant associations to genetic map regions. The correlation between MAD value and probability of genetic association for each bin was examined, using Pearson's correlation coefficient, in order to validate the usefulness of MAD values as a method of prioritization.

### Genetic analysis of quantitative data derived for NMR bins

The genetic marker data and associated genetic maps for seven linkage groups (LG) of the three recombinant inbred pea populations (JI 281 × JI 399; JI 15 × JI 399; JI 15 × JI 1194) which formed the basis for this study are available as Supplementary Data (Supplementary File S1, with the genetic map data available as Supplementary File S1–Figures M1–M9). Briefly, the genetic markers determined were based on gene-specific polymorphisms, as well as sequence-specific amplified polymorphic markers based on the retroelement PDR1 (Knox et al., 2009). Linkage analysis was carried out for three sets of RILs, and genetic maps obtained by ordering all available markers and determining their relative positions using THREaD MAPPER, a web-based software developed at JIC (Cheema et al., 2010), which can be accessed at http://threadmapper.org/threadmapper. The linkage maps generated were used to draw genetic map charts for assessing genetic loci associated with quantitative variation in NMR signals, which can be visualized across the NMR spectrum for all datasets as movies at http://www.threadmapper.org/qdips. Additionally, genetic markers associated with groups of NMR signals were analyzed in Excel, based on *p*-values as described below.

For high-throughput genetic analysis of quantitative trait data, single marker analyses were performed for 12 data sets (RIL population, year and location), using the linkage maps generated and the NMR bin quantitative signals as phenotypic data (968 and 990 bins for year 1 and year 2, respectively). A programme (available on request) was developed in house to generate *p*-values from Student's *t*-tests between RIL and maternal/paternal alleles. For most subsequent analyses, *p*-values were transformed to -log_10_ to enable map charts to be plotted for visual investigation.

**Figure 1:**
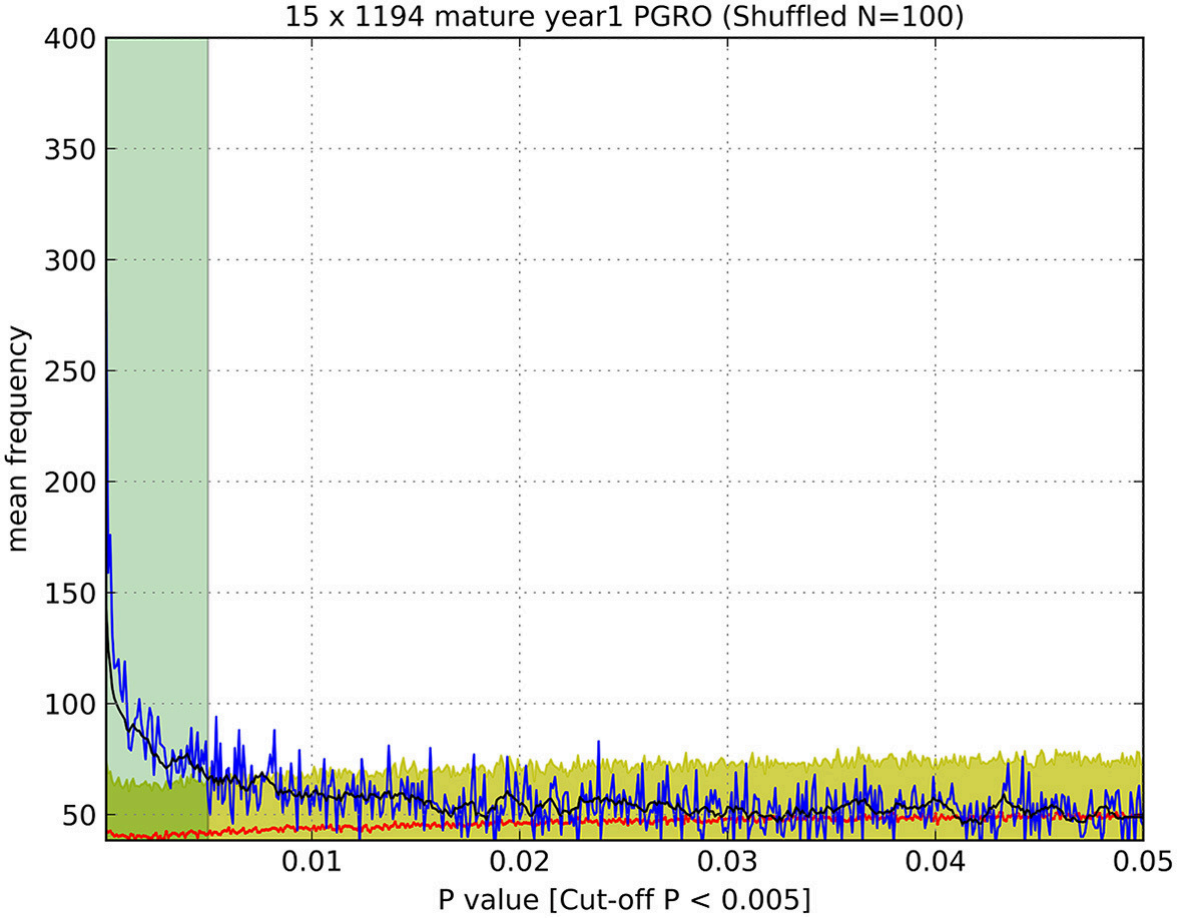
Determination of significance values (as listed in Table 1). An example of a plot of the observed number of *p*-values (blue line) of a given value (<0.05) compared to the number of *p*-values of that magnitude from randomized data (red line), which enabled the estimation of a cut-off value above which *p*-values were indistinguishable from random values. Black line: average of ten successive intervals, Yellow shadow: error (±3 S.D.) of 100 times resampled data, Green shadow: cut off point of *p*-values above the significance threshold. Data were determined for JI 15 × JI 1194 RILs, mature seeds, year 1, PGRO location.

Significance thresholds were determined for all datasets. To minimize the impact of false positive signals generated by multiple *t*-tests, the significance threshold for each dataset was determined individually. First, the frequency distribution of all *p*-values associated with markers for all bins within a dataset was plotted within the range of 0 < *p* ≤ 0.05 with intervals of 0.0001 (Figure 1, blue line) and the average of ten successive intervals generated to remove noise from raw data (Figure 1, black line). The data were subjected to randomization (100 times resampled) and the mean *p*-value frequency derived from the randomized data (Figure 1, red line) plotted, with error bars of ± 3 standard deviations (Figure 1, yellow shadow). A significance threshold for *p*-value was determined where the plot of the experimental dataset crossed over the upper error bar of the resampled (randomized) dataset as indicated (Figure 1, green shadow).

## Results

### Metabolite analysis

NMR spectra were acquired and peaks identified by adaptive binning (see section Materials and Methods, Davis et al., 2007) which allowed clear separation of peaks, and therefore calculation of peak areas, but with the consequence that the peaks did not coincide exactly between years. Some resonance frequencies included within a peak were known and thus bonds and compounds that contributed to the peak area could be identified. A list of correspondences and potential contributing compounds are given in Supplementary Tables 2, 3.

Peak areas were imported to Microsoft Excel Worksheets and, for a given data set (year and RIL population), the data were normalized so that each peak had an area of 100 and a standard deviation of 1. This meant that all peak areas were positive and that statistical analyses did not unduly emphasize variation in intense peaks and thus global analyses could be applied to the whole data set.

For a given population of RILs, the peak areas for each individual were available and the difference between the mean score for the lines with contrasting alleles was calculated. The expectation for each genotype is that the mean is 100 and the standard deviation is 1. The expected value of the difference between the two means is therefore 0 and given the number of individuals of each genotype a Student's *t*-test statistic can be generated. A related test calculated the mean absolute difference (Mean of Absolute Deviation from the mean, MAD) of the peak areas; this is greatest if there are two data subsets, one greater than 100 and the other less than 100. Example heat maps of MAD values in relation to the NMR spectrum are shown in Supplementary Figure 1, where regions of the spectrum showing consistently high variation are apparent. MAD values and the *t* statistic were well-correlated (Supplementary Figure 1).

The *t*-tests performed provided a probability value (*p*) for the two means being different from each other, but this is seriously confounded by multiple testing (ca. 1,000 bins and 790 markers). We therefore examined the frequency distribution of *p* as compared to randomized data in order to identify a threshold significance value for *p* (Figure 1).

### Genetic map based analysis

**Figure 2:**
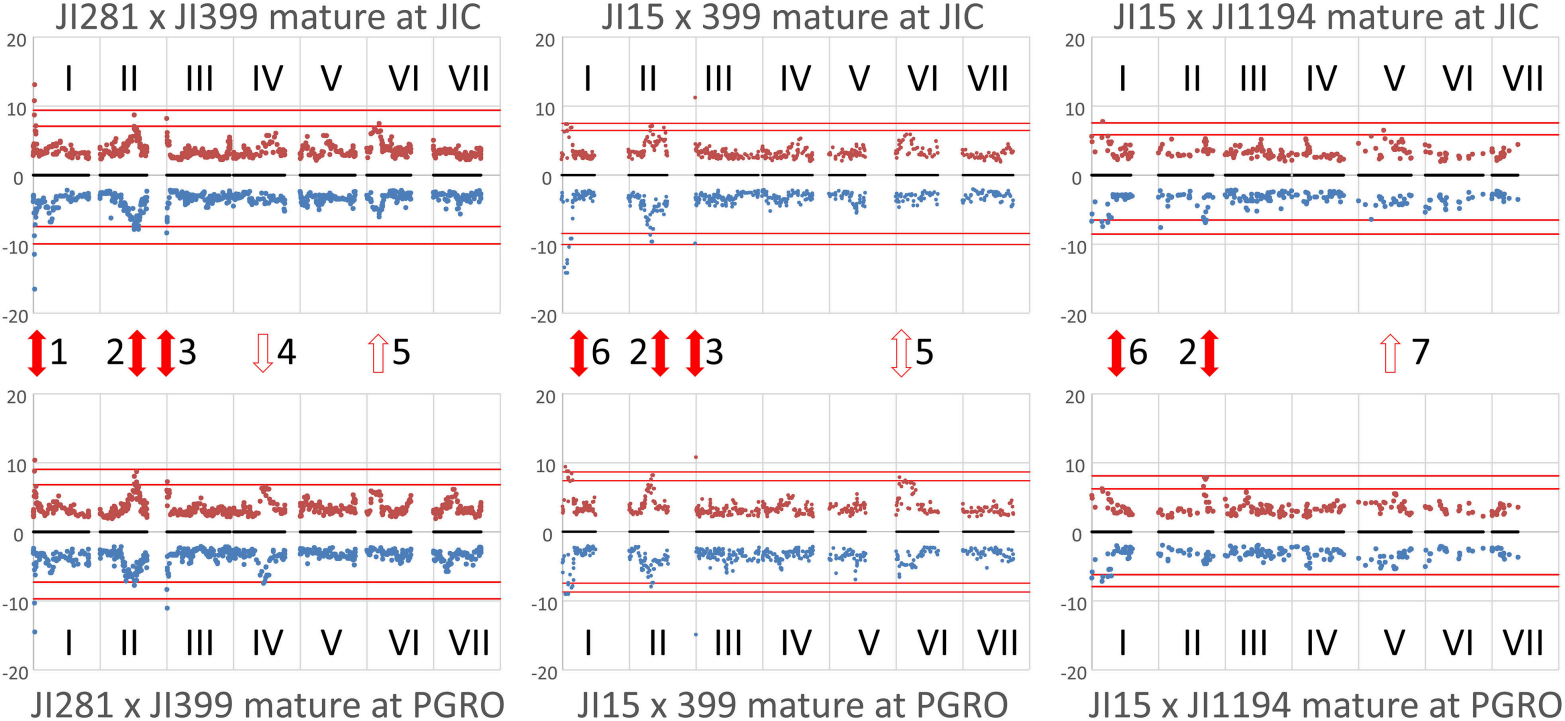
Locating the genomic regions with the most contrasting alleles. Genetic maps (linkage groups identified by roman numerals, I–VII) of the three populations analyzed (left to right JI 281 × JI399, JI 15 × JI 399, JI 15 × JI 1194) grown at JIC **(Top)** and PGRO **(Bottom)** are shown and the common logarithm of minimum *p*-value [–log_10_ (*p*)] for each marker is plotted (in blue, below the map for year 1 data, and −1 times this value in brown above the map for year 2 data). The red lines correspond to +3 and +5 standard deviations of all *p*-values for that population and location. Solid red arrows indicate marker-NMR resonance associations that are consistent between years, open arrows indicate those that reach the threshold value in 1 year, all numbered for reference in the text.

The determination of cut-off *p*-values generated large numbers of “significant” associations (Table 1). This suggested that, for most NMR bins, some genetic marker(s) could explain a component of their variation. While this is of theoretical interest, it does not focus attention on specific marker/metabolite associations. An alternative approach was taken where the minimum *p*-value for each marker was plotted against the genetic map of each RIL population (Figure 2). This identified those regions of the genetic map with the most significant effect on the metabolite profile and, once these had been identified, the NMR bin most affected by that marker could be identified.

**Table 1:** Summary of pea recombinant inbred populations for which metabolite data were collected for mature seeds in 2 years and two locations.

**Dataset**	**Significance threshold (*p*-value)**	**Number of significant marker bin associations**
JI 281 × JI 399, Y1, JIC	0.0073	18,040
JI 281 × JI 399, Y1, PGRO	0.0136	36,549
JI 281 × JI 399, Y2, JIC	0.0215	54,849
JI 281 × JI 399, Y2, PGRO	0.0071	19,261
JI 15 × JI 399, Y1, JIC	0.016	25,721
JI 15 × JI 399, Y1, PGRO	0.0168	27,136
JI 15 × JI 399, Y2, JIC	0.0001	398
JI 15 × JI 399, Y2, PGRO	0.0023	4,002
JI 15 × JI 1194, Y1, JIC	0.0035	3,012
JI 15 × JI 1194, Y1, PGRO	0.005	4,636
JI 15 × JI 1194, Y2, JIC	0.0018	1,392
JI 15 × JI 1194, Y2, PGRO	0.0003	401

*The significance threshold (see Figure 1) determined for every dataset [population identity, year (Y1 or Y2) and location of plant growth (JIC or PGRO)] is listed, together with the number of markers for which significant quantitative variation in NMR bin signal intensities was determined*.

The plot identifies regions of the genetic map that have important effects on the metabolite profile. It should be realized that these are regions of relative importance because the low *p*-values are included in the estimation of the standard deviations. If one of the most extreme peaks was missing, then the standard deviations used to estimate significance would be of a lower value. Those associations that are consistent between years are symmetrically reflected about the genetic map and equivalent positions on the genetic maps can be seen. The seven regions identified as peaks on the map (Figure 2) are discussed below.

#### Peak 1 and peak 6

These peaks are associated with a segment at the top of linkage group (LG) I characterized by the microsatellite marker PC20 and the gene encoding a small subunit of AGPase (Aubert et al., 2006). The classical locus *D* also maps in this region (Ellis and Poyser, 2002) and this locus is known to regulate the pattern of anthocyanin deposition. The resolution of the map is insufficient to identify a single causative allelic difference. However, although the peak assignments for this region (peaks 1 and 6) in the three populations at the two sites do not coincide exactly (Figure 2), the region includes some common genetic markers; the associated compounds are listed in Table 2. Note that these markers identify only the most significant associations, so a lack of replication does not mean that a shared association does not occur. One peak, and compound, implicated more than once is the flavanone glycoside hesperidin. The *p*-value data for the additional peaks identified for hesperidin, as shown in Supplementary Figure 2 for the JI 281 × JI 399 population (JIC, years 1 and 2), illustrate the consistency of this association. All but one of the bins that include a resonance assigned to hesperidin behave co-ordinately across the genetic map. This is consistent with the signals being derived from variation in the abundance of hesperidin or a closely related compound. The lack of significance for one bin (and an equivalent resonant range in both years) could be explained by a contribution from an additional signal in that bin from a compound that does not co-vary in abundance with hesperidin. Here, additional resonances are associated with aromatic compounds, tryptophan and its catabolite tyramine. Taken together, these signals suggest that there is allelic variation in this LG I region that alters the regulation of compounds closely related to the anthocyanin pathway, and the *D* locus may therefore be implicated.

**Table 2:** Peak 1 and Peak 6 resonances and associated NMR bin data.

**Population**	**Location**	**Year**	**Bin**	**ID comment**	**Bin range ppm**	**Compound ppm**
JI 281 × JI 399	JIC	1	**196** 199	**Hesperidin** Aromatic	7.106494107–7.096238809 7.09111116–7.085983511	7.106494 7.085984
		2	209 210 216	Aromatic, unknown Unknown Aromatic, unknown	7.139396521–7.130850439 7.130850439–7.125722791 7.089829248–7.077010126	7.13726 7.084702
	PGRO	1	**196**	**Hesperidin**	7.106494107–7.096238809	7.106494
		2	214	**Hesperidin**	7.107776019–7.09111116	7.106494
JI 15 × JI 399	JIC	1	**166** **182** 184 206 207	Aromatic, tentative **Naringin** Aromatic, tentative **Tyramine** **Tyrosine multiplet 3,5** Unassigned Aromatic, tentative **Chlorogenic acid**	7.359458121–7.347066302 7.237249155–7.224002728 7.219729688–7.209474390 7.050089969–7.041543887 7.041543887–7.031715894	7.359458 7.224003 7.2127 7.031716
		2	243	Unknown		6.9415
	PGRO	1	**166** **182** 219	Aromatic, tentative **Naringin** Aromatic, tentative **Tyramine** Unassigned	7.359458121–7.347066302 7.237249155–7.224002728 6.945400467–6.932581348	7.359458 7.224003 6.9415
		2	200 201 228	**Tyrosine multiplet 3,5** Aromatic, unknown Unassigned	7.198791788–7.186399969 7.186399969–7.178708496 7.042398495–7.03342511	7.1898 7.183409
JI 15 × JI 1194	JIC	1	166 183 **192**	Aromatic, tentative **Naringin** Tyrosine **Unassigned**	7.359458121–7.347066302 7.224002728–7.219729688 7.126150095–7.122304358	7.359458 7.21973
		2	196	Aromatic, unknown	7.237676459–7.226993857	7.237249
	PGRO	1	**182** 194 217	Aromatic, tentative **Tyramine** Unassigned Unassigned	7.237249155–7.224002728 7.110339844–7.108203323 6.966765674–6.948818903	7.224003
		2	**228**	**Unassigned**	7.042398495–7.03342511	

*Summary provides the population identity, year and location of plant growth (JIC or PGRO), NMR bin number and range, and compound information. In bold are the bin numbers and/or compound data that were identified consistently*.

#### Peak 2

This is a broad peak on LG II (Figure 2) and has its highest significance value in the population JI 281 × JI 399 associated with the classical gene *A*, which regulates anthocyanin biosynthesis and corresponds to a gene encoding a bHLH transcription factor (Hellens et al., 2010). The peak resonances are listed in Table 3; most of the bins are in the aromatic region of the spectrum, two in the sugar range and one in the expected range for aliphatic amino acids. The distribution of year 1 and year 2 *p*-values for the bin tentatively assigned as the flavonoid naringin in the JI 281 × JI 399 RILs (JIC location) are shown in Supplementary Figure 3 and show no significant *p*-value associated with *A*. Surprisingly although *A* and *a* segregate in all three populations analyzed, no signal for an anthocyanin was detected in this analysis. Kirby et al. (2013) have undertaken an NMR analysis of anthocyanins in *Rhus typhina*, identifying profiles with multiple resonances, and so it was expected that signals from pea anthocyanins might be similarly scattered throughout the NMR spectrum. The resonances identified by these authors as corresponding to aglycones can be aligned with the bins we defined in this group. These results are shown in Supplementary Figure 4, and suggest that it is likely that some *A*-regulated anthocyanins are detected by this analysis of mature seeds.

**Table 3:** Peak 2 resonances and associated NMR bin data.

**Population**	**Location**	**Year**	**Bin**	**Comment**	**Bin range ppm**	**Compound ppm**
JI 281 × JI 399	JIC	1	309 910	Unassigned Unassigned	6.252740561–6.231375357 0.881101007–0.869136493	
		2	432	Unassigned	5.709637079–5.698527173	
	PGRO	1	250	Unassigned	6.720211221–6.706110187	
		2	249 569 570	Unassigned Unknown Unknown	6.891560156–6.883868683 4.319616916–4.312780051 4.312780051–4.304661273	4.3151 4.3089
JI 15 × JI 299	JIC	1	299	Aromatic, unknown	6.371958398–6.34589285	6.371958
		2	**354**	**Unassigned**	6.289488712–6.271969244	
	PGRO	1	157	Unassigned	7.450046585–7.448337369	
		2	336	Aromatic, unknown	6.372385703–6.354866235	6.371958
JI 15 × JI 1194	JIC	1	307	Aromatic, tentative chlorogenic acid	7.450046585–7.448337369	
		2	n/a	n/a	n/a	
	PGRO	1	n/a	n/a	n/a	
		2	**354**	**Unassigned**	6.289488712–6.271969244	

*Summary provides the population identity, year (Y1 or Y2), location of plant growth (JIC or PGRO), NMR bin number and range, and compound information. In bold is the bin number identified more than once*.

#### Peak 3

Peak 3 corresponded to the top region of LG III in two of the three populations analyzed (Figure 2). This peak, close to the *rb* locus, may be considered an artefact due to there being very few *RbRb* genotypes within these two populations, where the vining genotypes selected for analysis were *rb* mutant lines (see section Materials and Methods). The *rb* mutation is a consequence of a nine-base pair deletion in the gene encoding the large subunit of AGPase (Rayner et al., 2017), which maps close to the top of LG III. It is noteworthy that this peak is missing from the JI 15 × JI 1194 population where all individuals are *RbRb*. Nevertheless, it is of interest that variation in metabolite profiles reflected the status of the *rb* locus.

#### Peak 4

A peak in the JI 281 × JI 399 population in the middle of LG IV for plants grown at PGRO is seen for both years (Figure 2). The peak of this value corresponding to bin 681 in JI 281 × JI 399 (Y1, PGRO) was not assigned to a known compound.

#### Peak 5

For both the JI 281 × JI 399 and JI 15 × JI 399 populations a peak can be seen in LG VI (Figure 2). The significant signals are for bin 490 in year 2 in JI 281 × JI 399 (JIC) and in JI 15 × JI 399 (PGRO), which was not assigned to a known compound but there is a resonance noted at 5.1052 ppm.

#### Peak 7

This corresponds to bin 879 in year 1 in the JI 15 × JI 1194 population and corresponds to an unidentified compound with a resonance at 1.1974 ppm.

### Compound based analyses

Using the genetic map as a way of identifying interesting compound/marker associations identified those regions of the map which had the most profound effect on the metabolite pool. However, this approach was limited because the association between NMR bins and known compounds within those bins was poorly established. It did suggest, however, that there are regions of the genome that have a major impact on the seed metabolome and which require further characterization, for example using 2D NMR or complementary analytical methods. The extent of this genetically controlled variation was revealed to be greater than initially expected, compared to analysis of leaf metabolomes (Charlton et al., 2008). A complementary approach was to examine variation associated with priority compounds, or classes of compounds, for which NMR resonances have been established.

Here we need to consider two problems. The first is that any bin contains more than one resonance frequency and so the signal intensity may reflect the abundance of more than one compound. The second problem is that any particular resonance may derive from more than one molecule, for example if the molecules differ at remote positions. One way to overcome this is to consider the behavior of the signals from molecules with many assigned resonances which might be expected to behave co-ordinately. Several amino acids fulfil these criteria and are discussed below.

#### Isoleucine

There are 23 bins that report the intensity of resonances from isoleucine in year 1 and year 2 data (see Supplementary Table 3). If these all report variation in the abundance of the same compound, then the *p*-values for each marker should be strongly correlated. Comparing the year 1 and year 2 data for JI 281 × JI 399 grown at JIC (Supplementary Table 4A), this is clearly not the case. The highest correlation coefficient is 0.344 (year 1 bins 795 and 904 vs. year 2 bin 977), and the lowest is −0.377 (year 1 bin 848 vs. year 2 bin 915). In contrast, comparison of the *p*-values for bins assigned to isoleucine within either year 1 or year 2 were highly correlated, with correlation coefficients reaching 0.97 in year 1 and 0.99 in year 2 (Supplementary Table 4B). This suggests that different bins are in fact reporting on co-varying determinants of the NMR signal, consistent with reporting on the same compound (or set of compounds). However, not all correlations were high even within an assignment class (defined in terms of the source of the NMR resonance in Supplementary Table 3). This is consistent with some bins reporting on resonance due to similar bonds in related (but different) compounds, or interference from resonances generated from different compounds that fall within the same bin.

**Figure 3:**
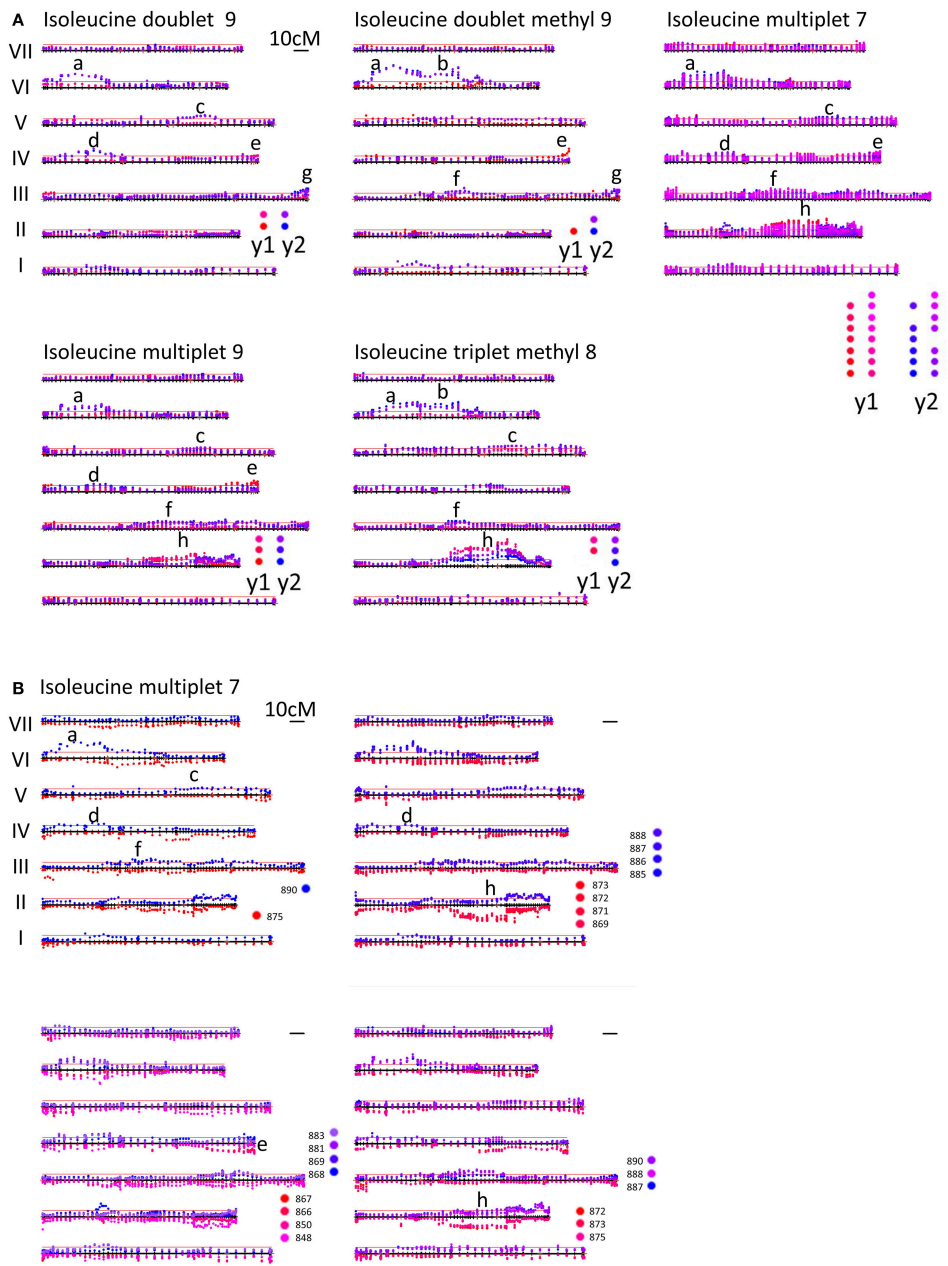
**(A)** Mapping variation attributed to isoleucine. LG I–VII of the genetic map of the JI 281 × JI 399 RIL population are displayed horizontally from bottom to top for each isoleucine signal. The –log_10_ (*p*)-values for each marker are plotted above the genetic map for NMR resonances associated with different parts of the isoleucine molecule. Different bins are plotted with a slightly different color, those from year 1 in red and those in year 2 in blue. Overall, the identified peaks (labeled a–h) are different between years, with the exception of peak h. The multiple bins assigned to isoleucine multiplet 7 are deconvoluted in **(B)**. Mapping variation attributed to isoleucine multiplet 7. LG I–VII of the genetic map of the JI 281 × JI 399 RIL population are displayed horizontally from bottom to top. The –log_10_ (*p*)-values for each marker are plotted above the genetic map for year 2 data and below the genetic map for year 1 data. The color coding for the various bins is indicated adjacent to the relevant map. The bin groupings are according to the correlations given in Supplementary Table 4. The peaks (labeled a–h) correspond to those identified in **(A)** for isoleucine multiplet 7. The red lines above the map correspond to 3 SD units for the variation in *p*-values.

The corresponding bins have different ppm ranges in different years; this may mean that the partitioning of signals is different between the data sets from the 2 years which may be more important than environmental effects on the biological samples. Indeed, the average of the correlation coefficients comparing overlapping bins between years is −0.024 ± 0.140 (*n* = 21), while the average of the correlation coefficient comparing non-overlapping bins between years is −0.027 ± 0.110 (*n* = 209), which would be consistent with the interpretation that overlapping bins are no more closely related than different bins that contain a resonance from the same compound; in other words, there are confounding signals within a bin. Partitioning these signals along the genetic map is therefore a useful way of dissecting out commonalities across the bins as shown in Figure 3. Although some peaks are consistent between Figures 3A,B, no peaks are consistent between years in Figure 3B, consistent with the suggestion that the bins are not comparable between years.

#### Leucine

Three bins were assigned to leucine in the year 1 and year 2 data, with details provided in Supplementary Table 5 and Supplementary Figure 5. These bins are close to one another and illustrate the way bins correspond between years. The data also highlight how adaptive binning can result in differences in the distribution of resonances among bins between the year 1 and year 2 datasets. (For example, the year 1 bin 903 includes the leucine resonance at 0.9494 ppm and the isoleucine resonance at 0.9584 ppm, but these are in separate bins in the year 2 data).

**Figure 4:**
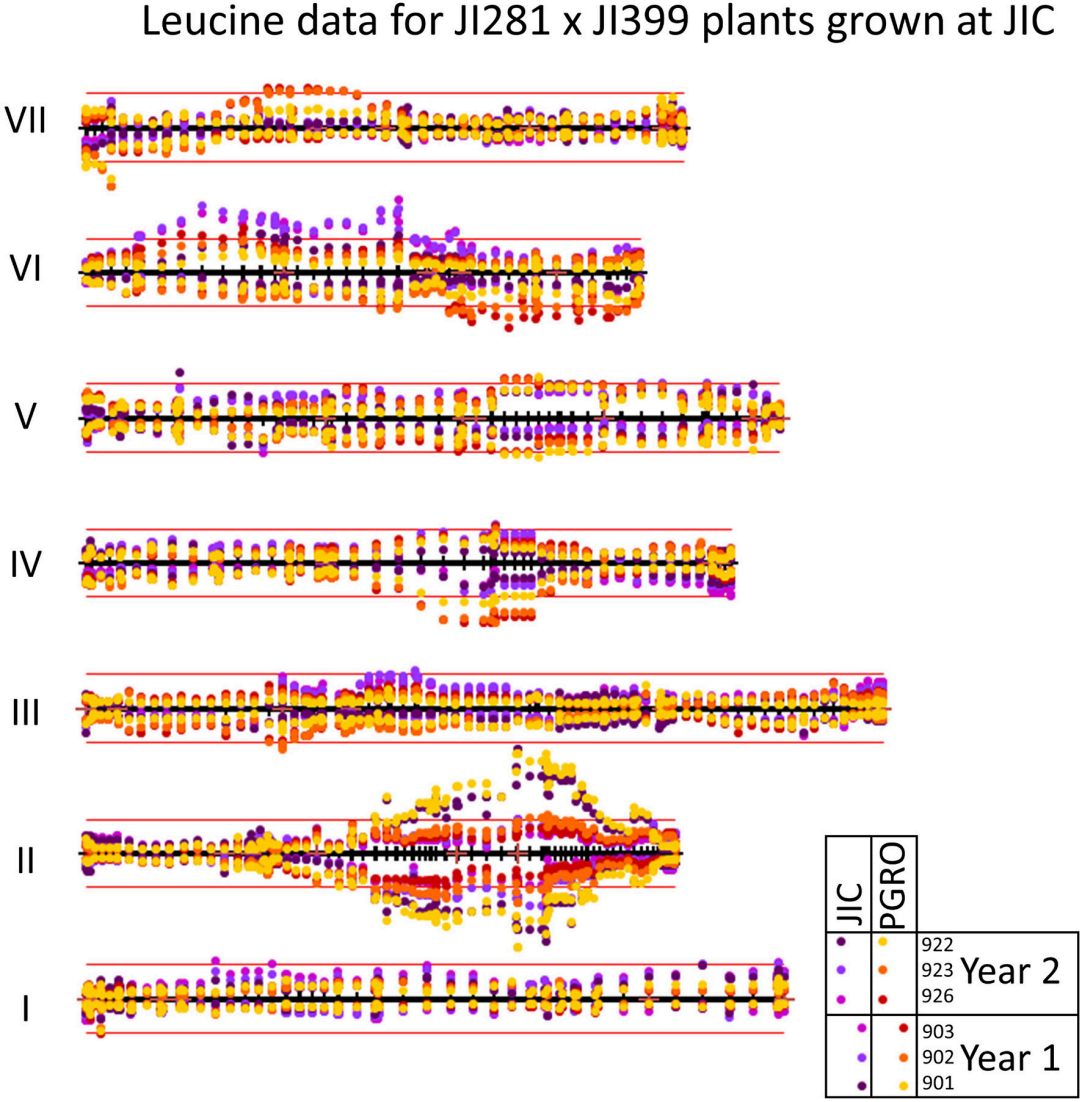
Mapping variation attributed to leucine. The seven linkage groups (I–VII) of the JI 281 × JI 399 genetic map are displayed horizontally from bottom to top. The –log_10_ (*p*) values for each marker are plotted above the genetic map for year 2 data and below the genetic map for year 1 data. The color coding for the various bins is indicated adjacent in the boxed section. The bins that contain the same resonance in year 1 and year 2 are given the same color. The red lines above and below the map correspond to 3 SD units for the variation in *p*-values for year 2 and year 1, respectively.

The distribution of –log_10_ (*p*) values for leucine is shown on the genetic linkage map in Figure 4. Here the reflection of the pattern above and below the map shows the consistency of the data between years and sites, which is particularly noticeable on LG II in the region of the classical genetic marker *A*. The correlations among leucine associated bins for two years are given in Supplementary Table 6. The correlations between sites and years for LG II are given in Table 4, where the most different site/year combination is JIC in year 2. Remarkably, the strongest and most consistent signal is coincident with the *A* locus. The direction of this effect shows that the allele *a* is associated with an increase in signal intensity (Supplementary Figure 6), implying a role for this locus in regulating compounds beyond anthocyanins.

**Table 4:** Linkage group II correlation coefficients of *p*-values among sites and years for leucine related NMR signals in the JI 281 × JI 399 RILs (Year 1, Year 2) across two sites (JIC, PGRO).

	**JIC Y1**	**JIC Y2**	**PGRO Y1**	**PGRO Y2**
JIC Y1		0.481	0.692	0.711
JIC Y2	0.481		0.580	0.680
PGRO Y1	0.692	0.580		0.719
PGRO Y2	0.711	0.680	0.719	

*Shading intensity is proportional to the value of the correlation coefficient*.

### The JI 281 × JI 399 recombinant inbred population

**Figure 5:**
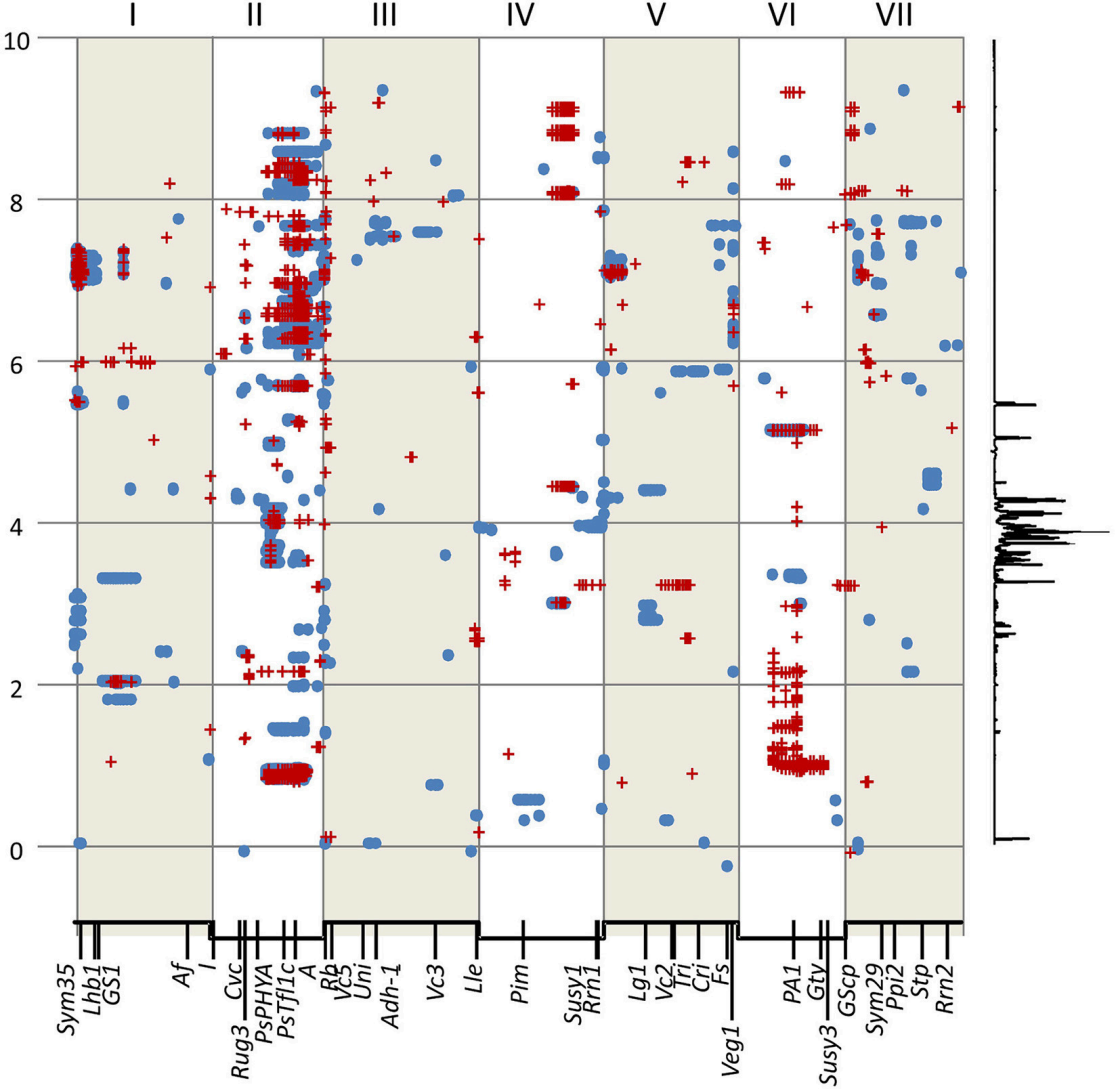
Significant genetic marker-NMR bin associations in the JI 281 × JI 399 RILs. The x axis represents a concatenated genetic map for LG I–VII in numerical order. Below the axis several reference genetic loci are indicated. The y axis represents the NMR spectrum in ppm. To the right of the graph a representative trace of the NMR spectrum is shown vertically to indicate relative signal intensity. The blue spots represent the upper and lower bounds of a selected year 1 bin and the red crosses are the upper and lower bounds of selected year 2 bins (both JIC location). Bins were selected that had –log_10_ (*p*) values greater than the mean plus 5 SD units.

Focussing on a single RIL population limits the analysis to a pair of alternative alleles, and we have shown above that the least correlated pair is the year 1 and year 2 data for the population JI 281 × JI 399 grown at JIC. We therefore examined these data sets and filtered according to the −log_10_ (*p*) values, selecting only marker/bin associations having a −log_10_ (*p*) value greater than 5 standard deviations from the mean of all values. For both years, this is a more stringent selection than using the *p*-values obtained using data randomization (see above). These data are summarized in Figure 5. Where there is correspondence in the identification of a marker/bin association at this level of stringency, the two types of symbol are coincident, whereas regions unique to a given year are indicated by the presence of a single symbol type (Figure 5). The resonance signals for all the bins where a compound has been identified are listed in Supplementary Table 7 and the compounds affected are listed in Table 5.

**Table 5:** Summary of compounds identified which differed in JI 281 × JI 399 RILs with high significance.

**Compounds identified in Years 1 and 2**			**Year 1 only**	**Year 2 only**
Alanine	Isoleucine	Sucrose	Aspartic acid	Phenylacetic acid
Arginine	Leucine	Trigonelline	Delphinidin	
Chlorogenic acid	Myoinositol	Tyramine	(or hesperidin)	
Folic acid	Naringin	Tyrosine	Dodecenic acid	
GABA	*p*-coumarate	Valine	Glutamine	
Glutamate	Phenylalanine	Verbascose	Methyl maleic acid	
Glutathione	Raffinose			
Hesperidin	Rutin			
Homoserine	Stachyose			

#### Raffinose and related oligosaccharides in the JI 281 × JI 399 population

**Figure 6:**
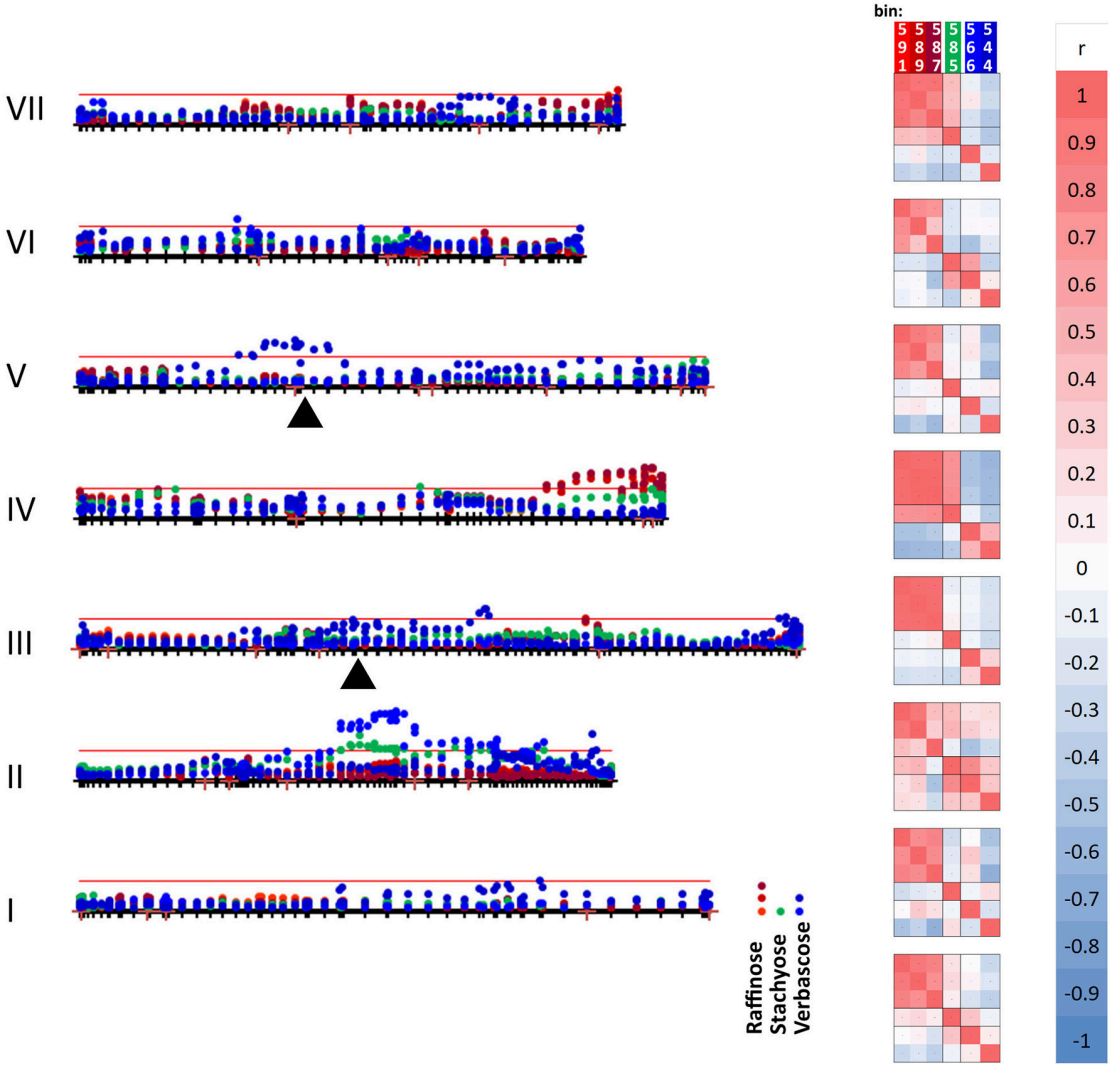
Mapping variation attributed to oligosaccharides. The seven linkage groups (I–VII) of the JI 281 × JI 399 genetic map are displayed horizontally from bottom to top. The –log10 (*p*) values for each oligosaccharide bin/marker are plotted above the genetic map (year 1, JIC). The threshold value for *p* determined from the randomization and *t*-test is indicated by the red horizontal line. The positions of raffinose synthase and stachyose synthase genes on linkage groups III and V are indicated by black triangles. The three bins assigned to raffinose (591, 589, and 587) are indicated in shades of red, stachyose (585) in green, and the two verbascose bins (566 and 544) in blue. On the right of the genetic map, a correlation analysis of the *p*-values for each bin within each linkage group is indicated, together with a color scale for the correlation range −1 to +1. At the bottom of the correlation scores, the correlation between the bins for the whole linkage map is given.

The raffinose family of oligosaccharides (RFOs) are among the list of compounds in Table 5. These three (raffinose, stachyose, verbascose) are related in terms of their biosynthesis (Peterbauer et al., 2002). One of the enzymes involved (raffinose synthase, Rfs) shows genetic variation that maps approximately centrally on LG III (close to PSAB124, PSAA491 and PSAC18 markers in the JI 281 × JI 399 population; close to agpS1_SNP3 on LG III in Iglesias-García et al., 2015). The gene encoding a second enzyme of this pathway, stachyose synthase (Sts), has been mapped to LG V in another cross (cv. Princess × JI 185, not used in this study). It is therefore of interest to describe how allelic variation for those bins, which contribute to the set displayed in Figure 5 and are associated with only one of these compounds, is distributed on the genetic map. This is illustrated in Figure 6.

The graph (Figure 6) includes an association between one of the verbascose-related bins (566) and the location of stachyose synthase on linkage group V. Overall, the correlation between the two verbascose bins (566, 544) is low, likely due to additional resonance signals. However, the correlations of bin 566 with the others assigned uniquely to RFOs suggests that this set of bins is reporting on related compounds. The slight elevation of –log_10_ (*p*) values near the location of the raffinose synthase gene does not reach the threshold level. This is notable, as there are eight amino acid differences between the deduced raffinose synthases of JI 281 and JI 399, four of which are predicted to lie within the mature protein (Q216K, R253W, G329V, and M379V for JI 281 and JI 399, respectively). The most significant associations for this group of compounds are with regions of LG II (stachyose, verbascose) and LG IV (raffinose, stachyose) (Figure 6).

Genetic loci on different linkage groups are associated with effects on raffinose concentration (Figure 6). The three raffinose bins (591, 589, and 587) are generally well correlated for the whole map, where the lowest correlation is for bins 589 and 587 with *r* = 0.729. However, the analysis for individual linkage groups has a range of correlation values, as would be expected if there is some interference from additional resonances that are under distinct genetic control. The most variable pair is 589 and 587 and their minimum correlation (for LG II) is 0.298 and maximum is 0.980 (for LG IV). Nevertheless, the strong correlation among these three bins is consistent with them reporting reasonably well on the same or a related compound. The most striking feature of these correlations according to linkage group is the contrast between LG II and LG IV. For LG II most correlations are positive; three negative pairwise combinations involve bin 587, with the other raffinose related bins having a positive correlation to both stachyose and verbascose bins. Linkage group II is the least differentiated in terms of these bins (measured as the mean average deviation of the non-self-correlations. For LG I–VII, these are: I, 0.331; II, 0.254; III, 0.351; IV, 0.616; V, 0.307; VI, 0.301; VII, 0.420, and overall 0.299). In contrast, LG IV is the most differentiated, with raffinose and stachyose positively correlated, but these are negatively correlated with verbascose, consistent with an allelic difference in the final step of the pathway. This is also seen on the –log_10_ (*p*) plot toward the right-hand side of LG IV (Figure 6), where the color symbols are well separated, suggesting a difference in control of the early and late steps in the RFO pathway. Within this group of compounds, the most intense NMR signal was from stachyose (bin 585) and this showed the largest actual difference in signal intensity between the contrasting allelic states (higher with the JI 399 allele); the greatest percentage difference between the allelic classes was for bin 544 (verbascose) which was higher when associated with the JI 281 allele.

## Discussion

In this paper, we investigate the genetic control of significant metabolites in pea seeds and provide a framework for their analysis in association with genetic maker data. Two approaches were adopted to examine the extent to which genetic, rather than environmental, control was important in determining the metabolome of seeds derived from three mapping populations: a map-based and a compound-based analysis. Despite the difficulties in associating NMR bin resonances exclusively with specific compounds, the screens have identified classes of compounds that should be investigated further as well as regions of the genetic map that warrant further investigation in relation to the compounds that are affected. We conclude that:

1. There are many different metabolites for which their abundance, within seeds of the RILs studied, varies under genetic control.
2. The genetic control of these compounds is distributed throughout the genetic map, with some regions implicated in the control of diverse metabolites.

An association between anthocyanin/phenylpropanoid derivatives and the nature of the allele at the *A* locus on LG II provides an example where the associated gene is a strong candidate for the observed effect, based on knowledge of flower color and seed trait differences associated with *A*/*a*. However, the highly significant differences in the branched chain amino acids (leucine, isoleucine) also associated with this locus suggests a wider impact on amino acid metabolism. This may be explained by considering that the anthocyanins are derived from phenylalanine/ phosphoenolpyruvate, while leucine/isoleucine are derived from pyruvate. Therefore, a reduced flux from phosphoenolpyruvate to phenylpropanoids in *a* mutants may generate a higher flux from pyruvate and hence more leucine/ isoleucine. This hypothesis is in agreement with the directional change in these amino acids (Supplementary Figure 6). Although the pool of free amino acids is relatively small in mature seeds, in comparison with protein-derived amino acids, it is likely to represent a component of the seed metabolome which is significant to seed storage and early germination. Fait et al. (2006) showed that the metabolic preparation for germination and efficient seedling establishment is initiated during seed desiccation. Understanding the genetic control of such variation is therefore of academic as well as economic interest. Other work has highlighted the impact of single gene changes on the seed metabolome; metabolomic profiling of pea lines down-regulated for AGPase has demonstrated the widespread consequences for metabolism of changes to this single gene (Weigelt et al., 2009). Significant variation in relative amounts of amino acids and in polyamine metabolism was reported in a study of seeds from wild type and mutant pea lines, differing by the presence or absence of pea albumin 2 genes, normally expressed in seeds (Vigeolas et al., 2008).

The genetic loci associated with variation in RFOs are equally of interest, with some genetic control possibly attributed to genes encoding the major synthetic enzymes of the pathway (LG III and LG V), but a much higher level of significance implicating control by genetic loci on LG II and IV. The below threshold variation associated with the different *Rfs* genes in JI 281 and JI 399 is in agreement with the different *Rfs* alleles encoding proteins that do not differ greatly in functionality. Certainly, none of the variant Rfs regions are predicted to be of high relevance to protein function (using CODDLE and PARSESNP programmes). The association of verbascose variation with LG V and the *Sts* gene may be consistent with the JI 281 *Sts* allele progressing the galactosylation of RFOs further than its JI 399 counterpart. Transfer of a further galactinol residue to stachyose gives verbascose, a reaction which is probably catalyzed by a bifunctional stachyose synthase (Peterbauer et al., 2001). In combination, these loci (Figure 6) may be important for determining seedling vigor. In *Medicago truncatula*, seven of the 12 QTL for germination rate or post-germinative growth parameters co-located with sucrose/RFO QTL (Vandecasteele et al., 2011). A significant negative correlation was also found between seed vigor traits and sucrose: RFO ratio and, in addition, 80% of the variation in the stachyose: verbascose ratio co-located with a stachyose synthase gene. The genetic control of RFOs is of additional interest, given their involvement more generally in abiotic and biotic stress responses (Cao et al., 2013; Nakabayashi and Kazuki, 2015).

Further development of the framework presented here for association of NMR resonances and genetic variation could include two-dimensional NMR on the contrasting genotypes, focussing on the resonances identified as being significant. Additionally, HPLC and/or GC-MS could supplement these analyses. The identification of candidate genes implicated in the genetic regions highlighted by this work could be accelerated by using the fast neutron mutant population, which has been developed for pea in one of the genetic backgrounds studied here (*rb* mutant) and where large genomic regions have been shown to be deleted (Domoney et al., 2013). Deletions could be positioned with respect to the genetic map and (when available) the genome sequence of pea to identify a subset of fast neutron mutants in which the NMR signals could be compared. Mutants affected in the relevant signal would presumably carry a deletion in the gene of interest and therefore it could be identified. These approaches would be complementary to those presented by Luo (2015) for metabolite-based genome-wide association studies in plants.

## Conclusion

NMR analysis of genetically marked lines of pea has revealed genetic variation associated with sets of metabolites present in mature seeds. Some of this variation may be explained by few genetic loci, including variation in compounds related to aromatic amino acids, branched-chain amino acids, sucrose-derived metabolites, secondary metabolites and some unidentified compounds. Overall there is extensive variation within *r* or *rb* genotypes that has major implications for seed quality traits and may impact nutritional and/or organoleptic parameters. This variation is under the control of multiple loci distributed throughout the genome, presenting an array of possibilities for breeders. Our approach shows how the major genetic determinants of such variation can be identified and therefore managed within a breeding programme. The combined analysis thus presented provides a framework for the genetic analysis of the seed metabolome. The genetic marker datasets provided may be used in the further analysis of seed components that relate directly to seed storage and end-use quality traits.

## Author contributions

NE, CH, and CD conceptualized the research. NE, PK, ZS, GK, and CH performed genetic mapping and analyzed genetic map data. JD, MD, AC, and GV carried out the NMR and data analysis. CH, JC, and NE devised and performed the bioinformatic analysis of the NMR and genetic data; NE and CD drafted and finalized the paper.

### Conflict of interest statement

JD, AC, MD, and GV were employed by The Food & Environment Research Agency, a government agency (now the company Fera Science Ltd.). GK is employed by the company AMBIS Biotechnology Ltd. The remaining authors declare that the research was conducted in the absence of any commercial or financial relationships that could be construed as a potential conflict of interest.
