# Supplementary material for: NMR Metabolomics Defining Genetic Variation in Pea Seed Metabolites
Source: Front Plant Sci. 2018 Jul 17;9:1022. doi: 10.3389/fpls.2018.01022 (PMC6056766; doi:10.3389/fpls.2018.01022)
Supplement: Supplementary file 2 [file Table_2.docx]

### **Supplementary Table S2. Summary of resonances mapped in Figure S4**

| Aglycones assigned by Kirby et al., 2013 (ppm) | Start (ppm) | End (ppm) | Year 1 bin | Resonance | Compounds assigned (present study) |
| --- | --- | --- | --- | --- | --- |
| 4.01 | 4.011103372 | 4.005548419 | 583 | 4.0061 | Raffinose doublet of doublets, 24 |
| 4.03 | 4.048278827 | 4.025204407 | **580^a^** | 4.0273 | Unknown |
| 4.08 | 4.083745065 | 4.066225598 | 578 | 4.0706 | Unknown |
| 4.09 | 4.104255661 | 4.083745065 | 577 | 4.0873 | Raffinose multiplet 19, 9, 4 /stachyose multiplet 24, 19, 35, 4 |
| 6.68 | 6.684317679 | 6.676198901 | 254 |  |  |
| 6.82 | 6.821054984 | 6.810799686 | 238 |  |  |
| 6.88 | 6.881732162 | 6.869767648 | **225^a^** |  |  |
| 6.89 | 6.906515799 | 6.884723291 | **223^a^** | 6.8945 | Tyrosine multiplet 2,6 |
| 6.99 | 6.99154931 | 6.983003229 | 214 |  |  |
| 7.01 | 7.013769122 | 7.001377304 | 211 |  |  |
| 7.06 | 7.066327524 | 7.057354138 | 204 |  |  |
| 7.08 | 7.080001254 | 7.076582822 | 202 |  |  |
| 7.12 | 7.122304358 | 7.110339844 | 193 | 7.1223 | Hesperidin |
| 7.19 | 7.195800659 | 7.183408841 | 187 | 7.1834 | Aromatic, unknown |
| 7.37 | 7.370995331 | 7.359458121 | 165 | 7.3679 | Phenylalanine |
| 7.46 | 7.473975613 | 7.459019971 | 154 |  |  |
| 7.57 | 7.574819376 | 7.568409814 | 143 |  |  |
| 7.63 | 7.63293273 | 7.626523169 | 135 |  |  |
| 7.73 | 7.7346311 | 7.717966241 | 119 |  |  |
| 7.79 | 7.79018063 | 7.786334894 | 111 |  |  |
| 7.89 | 7.89914317 | 7.879914487 | 102 |  |  |
| 7.92 |  |  |  |  | (Not binned) |
| 8.03 |  |  |  |  | (Not binned) |
| 8.08 | 8.087584268 | 8.079038187 | **84^a^** |  |  |
| 8.95 |  |  |  |  | (Not binned) |
| 9.06 |  |  |  |  | (Not binned) |

**^a^ significant difference according to the allele (*A* or *a*) at the *A* locus**
