# Supplementary material for: NMR Metabolomics Defining Genetic Variation in Pea Seed Metabolites
Source: Front Plant Sci. 2018 Jul 17;9:1022. doi: 10.3389/fpls.2018.01022 (PMC6056766; doi:10.3389/fpls.2018.01022)
Supplement: Supplementary file 3 [file Table_3.docx]

### **Supplementary Table S3. Isoleucine resonances,** **corresponding ppm and bin numbers in the two datasets (Year 1, Year 2)**

| Year 1 | |  |  |  | Year 2 | |  |  |  |  |
| --- | --- | --- | --- | --- | --- | --- | --- | --- | --- | --- |
| bin | Start (ppm) | End (ppm) | Chemical shift (ppm) |  | bin | Start (ppm) | End (ppm) | Chemical shift (ppm) |  | Assignment |
| 885 | 1.145174927 | 1.129364676 | 1.1385 |  | 900 | 1.145602231 | 1.123382419 | 1.1385 |  | Isoleucine doublet 9 |
| 884 | 1.156712137 | 1.145174927 | 1.1525 |  | 899 | 1.157139441 | 1.145602231 | 1.1525 |  | Isoleucine doublet 9 |
|  |  |  |  |  |  |  |  |  |  |  |
|  |  |  |  |  | 918 | 1.014847183 | 1.006301102 | 1.0078 |  | Isoleucine doublet methyl 9 |
| 898 | 1.029802826 | 1.014847183 | 1.0219 |  | 915 | 1.028948218 | 1.0165564 | 1.0219 |  | Isoleucine doublet methyl 9 |
|  |  |  |  |  |  |  |  |  |  |  |
| 875 | 1.23149035 | 1.219525836 | 1.2265 |  | 890 | 1.231917654 | 1.226362701 | 1.2265 |  | Isoleucine multiplet 7 |
| 873 | 1.245164081 | 1.236617999 | 1.2407 |  | 888 | 1.244736777 | 1.236617999 | 1.2407 |  | Isoleucine multiplet 7 |
| 872 | 1.25029173 | 1.245164081 | 1.2473 |  | 887 | 1.25029173 | 1.244736777 | 1.2473 |  | Isoleucine multiplet 7 |
| 871 | 1.260974332 | 1.25029173 | 1.2547 |  | 886 | 1.267383893 | 1.25029173 | 1.2547 |  | Isoleucine multiplet 7 |
| 870 | 1.269520413 | 1.260974332 | 1.2642 |  |  |  |  |  |  | Isoleucine multiplet 7 |
| 869 | 1.274648062 | 1.269520413 | 1.2731 |  | 885 | 1.275929974 | 1.267383893 | 1.2731 |  | Isoleucine multiplet 7 |
| 868 | 1.282339536 | 1.274648062 | 1.2784 |  | 884 | 1.281912231 | 1.275929974 | 1.2784 |  | Isoleucine multiplet 7 |
| 867 | 1.290458313 | 1.282339536 | 1.2855 |  | 883 | 1.288749097 | 1.281912231 | 1.2855 |  | Isoleucine multiplet 7 |
| 866 | 1.301995523 | 1.290458313 | 1.2932 |  | 881 | 1.332334113 | 1.290031009 | 1.2932 |  | Isoleucine multiplet 7 |
| 865 | 1.336179849 | 1.301995523 | 1.3132 |  |  |  |  |  |  | Isoleucine multiplet 7 |
| 850 | 1.435741699 | 1.428904834 | 1.435 |  | 869 | 1.440442044 | 1.434887091 | 1.435 |  | Isoleucine multiplet 7 |
| 848 | 1.446851605 | 1.441723956 | 1.4441 |  | 868 | 1.447706213 | 1.440442044 | 1.4441 |  | Isoleucine multiplet 7 |
| 847 | 1.456252295 | 1.446851605 | 1.4511 |  | 867 | 1.454543079 | 1.447706213 | 1.4511 |  | Isoleucine multiplet 7 |
| 846 | 1.461807248 | 1.456252295 | 1.4603 |  | 866 | 1.46308916 | 1.454543079 | 1.4603 |  | Isoleucine multiplet 7 |
| 845 | 1.48616358 | 1.461807248 | 1.4652 |  | 865 | 1.468644113 | 1.46308916 | 1.4652 |  | Isoleucine multiplet 7 |
|  |  |  |  |  |  |  |  |  |  |  |
| 795 | 1.979272485 | 1.967307971 | 1.9717 |  | 802 | 1.978845181 | 1.966453363 | 1.9717 |  | Isoleucine multiplet 9 |
| 791 | 2.00704725 | 1.99551004 | 2.0035 |  | 799 | 2.00491073 | 1.997219257 | 2.0035 |  | Isoleucine multiplet 9 |
| 789 | 2.019866373 | 2.013456812 | 2.0176 |  | 797 | 2.020293677 | 2.013029507 | 2.0176 |  | Isoleucine multiplet 9 |
|  |  |  |  |  |  |  |  |  |  |  |
| 906 | 0.933232104 | 0.925113327 | 0.9287 |  | 929 | 0.931950192 | 0.925967935 | 0.9287 |  | Isoleucine triplet methyl 8 |
| 904 | 0.948187747 | 0.938787057 | 0.9436 |  | 927 | 0.947333139 | 0.940496274 | 0.9436 |  | Isoleucine triplet methyl 8 |
|  |  |  |  |  | 924 | 0.961434174 | 0.955879221 | 0.9584 |  | Isoleucine triplet methyl 8 |
