# Supplementary material for: NMR Metabolomics Defining Genetic Variation in Pea Seed Metabolites
Source: Front Plant Sci. 2018 Jul 17;9:1022. doi: 10.3389/fpls.2018.01022 (PMC6056766; doi:10.3389/fpls.2018.01022)
Supplement: Supplementary file 4 [file Table_4.docx]

### **Supplementary Table S4. Correlations between isoleucine resonances.** Bin numbers are given in the top row and first column.

#### **A. Correlations between years**

| Correlation between years (the diagonal set of correlations corresponds to bins with overlapping ranges in the two years) | | | | | | | | | | | | | | | | | | | | | | | |
| --- | --- | --- | --- | --- | --- | --- | --- | --- | --- | --- | --- | --- | --- | --- | --- | --- | --- | --- | --- | --- | --- | --- | --- |
| bin | 899 | 900 | 915 | 865 | 866 | 867 | 868 | 869 | 881 | 883 | 884 | 885 | 886 | 887 | 888 | 890 | 797 | 799 | 802 | 927 | 929 | Y1 | scale |
| 884 | -0.127 | -0.101 | -0.124 | -0.092 | -0.057 | -0.074 | -0.020 | -0.003 | 0.040 | -0.058 | -0.011 | 0.001 | -0.060 | 0.115 | 0.015 | 0.054 | -0.052 | -0.066 | -0.126 | 0.081 | 0.046 |  | -1.000 |
| 885 | -0.117 | -0.096 | -0.126 | -0.104 | -0.075 | -0.096 | -0.044 | -0.027 | 0.036 | -0.059 | -0.013 | -0.010 | -0.053 | 0.101 | 0.031 | 0.052 | -0.069 | -0.081 | -0.125 | 0.063 | 0.024 |  | -0.900 |
| 898 | -0.052 | -0.095 | -0.080 | -0.242 | -0.261 | -0.252 | -0.258 | -0.244 | -0.018 | -0.023 | -0.039 | -0.005 | -0.076 | -0.219 | -0.209 | -0.300 | -0.190 | -0.206 | -0.214 | -0.125 | -0.151 |  | -0.800 |
| 845 | -0.237 | -0.235 | -0.249 | -0.082 | -0.046 | -0.064 | -0.040 | -0.039 | 0.161 | -0.089 | -0.066 | -0.108 | -0.166 | -0.092 | 0.073 | 0.152 | -0.028 | -0.063 | -0.178 | 0.057 | 0.051 |  | -0.700 |
| 846 | -0.258 | -0.309 | -0.331 | -0.168 | -0.098 | -0.122 | -0.074 | -0.085 | 0.109 | -0.134 | -0.075 | -0.146 | -0.221 | -0.047 | 0.036 | 0.085 | -0.056 | -0.063 | -0.130 | 0.134 | 0.149 |  | -0.600 |
| 847 | -0.271 | -0.340 | -0.372 | -0.235 | -0.165 | -0.192 | -0.141 | -0.146 | 0.088 | -0.142 | -0.074 | -0.145 | -0.249 | -0.077 | -0.023 | 0.016 | -0.087 | -0.095 | -0.175 | 0.079 | 0.083 |  | -0.500 |
| 848 | -0.229 | -0.303 | -0.377 | -0.209 | -0.146 | -0.166 | -0.122 | -0.133 | 0.155 | -0.024 | 0.048 | -0.044 | -0.188 | -0.023 | -0.030 | -0.004 | -0.059 | -0.051 | -0.122 | 0.079 | 0.073 |  | -0.400 |
| 850 | -0.219 | -0.296 | -0.348 | -0.221 | -0.164 | -0.179 | -0.139 | -0.150 | 0.160 | -0.002 | 0.077 | -0.016 | -0.149 | 0.008 | -0.029 | -0.023 | -0.085 | -0.085 | -0.143 | 0.054 | 0.047 |  | -0.300 |
| 866 | 0.036 | 0.022 | 0.008 | 0.002 | 0.000 | -0.009 | 0.025 | 0.039 | -0.091 | -0.031 | -0.063 | 0.000 | 0.115 | 0.060 | 0.061 | -0.014 | -0.028 | -0.074 | -0.091 | -0.057 | -0.059 |  | -0.200 |
| 867 | 0.075 | 0.081 | 0.066 | 0.072 | 0.076 | 0.066 | 0.102 | 0.113 | -0.024 | 0.000 | -0.023 | 0.027 | 0.163 | 0.123 | 0.160 | 0.078 | 0.019 | -0.036 | -0.058 | -0.012 | -0.012 |  | -0.100 |
| 868 | 0.081 | 0.083 | 0.068 | 0.028 | 0.033 | 0.024 | 0.061 | 0.073 | -0.007 | 0.016 | 0.025 | 0.040 | 0.130 | 0.090 | 0.116 | 0.048 | -0.027 | -0.086 | -0.109 | -0.017 | -0.027 |  | 0.000 |
| 869 | -0.073 | -0.036 | 0.010 | -0.004 | 0.054 | 0.039 | 0.101 | 0.100 | 0.159 | 0.006 | 0.071 | 0.051 | 0.040 | 0.137 | 0.125 | 0.107 | -0.040 | -0.092 | -0.199 | 0.132 | 0.112 |  | 0.100 |
| 871 | -0.126 | -0.129 | -0.196 | -0.109 | -0.049 | -0.073 | -0.006 | 0.010 | 0.050 | -0.066 | 0.001 | -0.046 | -0.141 | 0.044 | -0.029 | 0.025 | -0.071 | -0.086 | -0.186 | 0.089 | 0.055 |  | 0.200 |
| 872 | -0.156 | -0.161 | -0.247 | -0.090 | -0.025 | -0.048 | 0.020 | 0.032 | 0.113 | -0.089 | -0.045 | -0.063 | -0.145 | 0.102 | 0.032 | 0.092 | -0.028 | -0.047 | -0.184 | 0.131 | 0.088 |  | 0.300 |
| 873 | -0.124 | -0.143 | -0.238 | -0.131 | -0.072 | -0.095 | -0.031 | -0.011 | 0.019 | -0.073 | -0.016 | -0.037 | -0.149 | 0.073 | -0.046 | 0.034 | -0.070 | -0.074 | -0.185 | 0.088 | 0.051 |  | 0.400 |
| 875 | -0.161 | -0.162 | -0.252 | -0.144 | -0.078 | -0.107 | -0.040 | -0.026 | 0.021 | -0.068 | -0.014 | -0.041 | -0.175 | 0.055 | -0.038 | 0.055 | -0.093 | -0.094 | -0.208 | 0.095 | 0.060 |  | 0.500 |
| 789 | -0.139 | -0.184 | -0.248 | -0.225 | -0.157 | -0.190 | -0.122 | -0.117 | 0.047 | -0.085 | -0.005 | -0.078 | -0.272 | -0.074 | -0.123 | -0.041 | -0.048 | -0.055 | -0.197 | 0.087 | 0.063 |  | 0.600 |
| 791 | -0.208 | -0.275 | -0.309 | -0.273 | -0.210 | -0.247 | -0.185 | -0.185 | 0.056 | -0.122 | -0.011 | -0.129 | -0.285 | -0.087 | -0.127 | -0.074 | -0.145 | -0.132 | -0.202 | 0.039 | -0.001 |  | 0.700 |
| 795 | -0.154 | -0.142 | -0.188 | 0.018 | 0.089 | 0.080 | 0.117 | 0.131 | 0.039 | -0.065 | -0.057 | -0.036 | -0.182 | 0.128 | -0.011 | 0.123 | 0.073 | 0.140 | 0.011 | 0.344 | 0.337 |  | 0.800 |
| 904 | -0.154 | -0.142 | -0.188 | 0.018 | 0.089 | 0.080 | 0.117 | 0.131 | 0.039 | -0.065 | -0.057 | -0.036 | -0.182 | 0.128 | -0.011 | 0.123 | 0.073 | 0.140 | 0.011 | 0.344 | 0.337 |  | 0.900 |
| 906 | -0.146 | -0.128 | -0.170 | 0.054 | 0.122 | 0.113 | 0.147 | 0.156 | 0.089 | -0.079 | -0.050 | -0.051 | -0.143 | 0.171 | 0.075 | 0.171 | 0.072 | 0.125 | -0.006 | 0.315 | 0.310 |  | 1.000 |
| Y2 |  |  |  |  |  |  |  |  |  |  |  |  |  |  |  |  |  |  |  |  |  |  |  |

### **Supplementary Table S4. Correlations between isoleucine resonances (continued)**

#### **B. Correlations within years**

Within year correlations ordered by assignment (Year 1, top; Year 2, bottom)

|  | | | | |  |  |  |  |  |  |  |  |  |  |  |  |  |  |  |  |  |  |  |
| --- | --- | --- | --- | --- | --- | --- | --- | --- | --- | --- | --- | --- | --- | --- | --- | --- | --- | --- | --- | --- | --- | --- | --- |
| bin | 884 | 885 | 898 | 845 | 846 | 847 | 848 | 850 | 866 | 867 | 868 | 869 | 871 | 872 | 873 | 875 | 789 | 791 | 795 | 904 | 906 |  | scale |
| 884 |  | 0.970 | 0.042 | 0.231 | 0.099 | 0.164 | 0.172 | 0.230 | 0.425 | 0.406 | 0.460 | 0.651 | 0.763 | 0.785 | 0.723 | 0.720 | 0.494 | 0.353 | 0.028 | 0.287 | 0.299 |  | -1.000 |
| 885 | 0.970 |  | 0.052 | 0.235 | 0.109 | 0.178 | 0.179 | 0.238 | 0.438 | 0.420 | 0.481 | 0.625 | 0.737 | 0.750 | 0.701 | 0.695 | 0.480 | 0.371 | 0.056 | 0.242 | 0.259 |  | -0.900 |
| 898 | 0.042 | 0.052 |  | 0.165 | -0.036 | 0.035 | -0.017 | -0.072 | 0.226 | 0.147 | 0.167 | 0.050 | 0.070 | 0.022 | 0.127 | 0.133 | 0.097 | 0.137 | 0.082 | 0.021 | -0.023 |  | -0.800 |
| 845 | 0.231 | 0.235 | 0.165 |  | 0.557 | 0.562 | 0.417 | 0.296 | 0.068 | 0.044 | 0.074 | 0.190 | 0.381 | 0.399 | 0.434 | 0.494 | 0.466 | 0.449 | 0.206 | 0.233 | 0.206 |  | -0.700 |
| 846 | 0.099 | 0.109 | -0.036 | 0.557 |  | 0.968 | 0.875 | 0.766 | -0.105 | -0.082 | -0.072 | 0.104 | 0.170 | 0.256 | 0.229 | 0.272 | 0.454 | 0.605 | 0.356 | 0.305 | 0.292 |  | -0.600 |
| 847 | 0.164 | 0.178 | 0.035 | 0.562 | 0.968 |  | 0.889 | 0.795 | -0.062 | -0.047 | -0.027 | 0.136 | 0.238 | 0.304 | 0.296 | 0.332 | 0.528 | 0.714 | 0.404 | 0.298 | 0.296 |  | -0.500 |
| 848 | 0.172 | 0.179 | -0.017 | 0.417 | 0.875 | 0.889 |  | 0.935 | -0.082 | -0.076 | -0.037 | 0.158 | 0.258 | 0.338 | 0.298 | 0.321 | 0.484 | 0.677 | 0.467 | 0.286 | 0.308 |  | -0.400 |
| 850 | 0.230 | 0.238 | -0.072 | 0.296 | 0.766 | 0.795 | 0.935 |  | -0.022 | -0.023 | 0.022 | 0.221 | 0.295 | 0.366 | 0.300 | 0.308 | 0.434 | 0.642 | 0.455 | 0.177 | 0.230 |  | -0.300 |
| 866 | 0.425 | 0.438 | 0.226 | 0.068 | -0.105 | -0.062 | -0.082 | -0.022 |  | 0.941 | 0.901 | 0.565 | 0.459 | 0.397 | 0.320 | 0.298 | 0.141 | 0.132 | -0.008 | -0.022 | 0.041 |  | -0.200 |
| 867 | 0.406 | 0.420 | 0.147 | 0.044 | -0.082 | -0.047 | -0.076 | -0.023 | 0.941 |  | 0.938 | 0.645 | 0.422 | 0.381 | 0.264 | 0.245 | 0.102 | 0.108 | -0.001 | -0.033 | 0.065 |  | -0.100 |
| 868 | 0.460 | 0.481 | 0.167 | 0.074 | -0.072 | -0.027 | -0.037 | 0.022 | 0.901 | 0.938 |  | 0.702 | 0.504 | 0.432 | 0.332 | 0.303 | 0.147 | 0.164 | 0.069 | -0.040 | 0.070 |  | 0.000 |
| 869 | 0.651 | 0.625 | 0.050 | 0.190 | 0.104 | 0.136 | 0.158 | 0.221 | 0.565 | 0.645 | 0.702 |  | 0.667 | 0.657 | 0.525 | 0.499 | 0.302 | 0.272 | 0.094 | 0.124 | 0.238 |  | 0.100 |
| 871 | 0.763 | 0.737 | 0.070 | 0.381 | 0.170 | 0.238 | 0.258 | 0.295 | 0.459 | 0.422 | 0.504 | 0.667 |  | 0.915 | 0.899 | 0.852 | 0.593 | 0.480 | 0.160 | 0.314 | 0.327 |  | 0.200 |
| 872 | 0.785 | 0.750 | 0.022 | 0.399 | 0.256 | 0.304 | 0.338 | 0.366 | 0.397 | 0.381 | 0.432 | 0.657 | 0.915 |  | 0.875 | 0.874 | 0.634 | 0.490 | 0.142 | 0.409 | 0.436 |  | 0.300 |
| 873 | 0.723 | 0.701 | 0.127 | 0.434 | 0.229 | 0.296 | 0.298 | 0.300 | 0.320 | 0.264 | 0.332 | 0.525 | 0.899 | 0.875 |  | 0.953 | 0.674 | 0.527 | 0.177 | 0.368 | 0.330 |  | 0.400 |
| 875 | 0.720 | 0.695 | 0.133 | 0.494 | 0.272 | 0.332 | 0.321 | 0.308 | 0.298 | 0.245 | 0.303 | 0.499 | 0.852 | 0.874 | 0.953 |  | 0.682 | 0.537 | 0.175 | 0.471 | 0.417 |  | 0.500 |
| 789 | 0.494 | 0.480 | 0.097 | 0.466 | 0.454 | 0.528 | 0.484 | 0.434 | 0.141 | 0.102 | 0.147 | 0.302 | 0.593 | 0.634 | 0.674 | 0.682 |  | 0.712 | 0.228 | 0.281 | 0.225 |  | 0.600 |
| 791 | 0.353 | 0.371 | 0.137 | 0.449 | 0.605 | 0.714 | 0.677 | 0.642 | 0.132 | 0.108 | 0.164 | 0.272 | 0.480 | 0.490 | 0.527 | 0.537 | 0.712 |  | 0.566 | 0.240 | 0.250 |  | 0.700 |
| 795 | 0.028 | 0.056 | 0.082 | 0.206 | 0.356 | 0.404 | 0.467 | 0.455 | -0.008 | -0.001 | 0.069 | 0.094 | 0.160 | 0.142 | 0.177 | 0.175 | 0.228 | 0.566 |  | 0.132 | 0.209 |  | 0.800 |
| 904 | 0.287 | 0.242 | 0.021 | 0.233 | 0.305 | 0.298 | 0.286 | 0.177 | -0.022 | -0.033 | -0.040 | 0.124 | 0.314 | 0.409 | 0.368 | 0.471 | 0.281 | 0.240 | 0.132 |  | 0.907 |  | 0.900 |
| 906 | 0.299 | 0.259 | -0.023 | 0.206 | 0.292 | 0.296 | 0.308 | 0.230 | 0.041 | 0.065 | 0.070 | 0.238 | 0.327 | 0.436 | 0.330 | 0.417 | 0.225 | 0.250 | 0.209 | 0.907 |  |  | 1.000 |
|  |  |  |  |  |  |  |  |  |  |  |  |  |  |  |  |  |  |  |  |  |  |  |  |

|  |  |  |  |  |  |  |  |  |  |  |  |  |  |  |  |  |  |  |  |  |  |  |  |
| --- | --- | --- | --- | --- | --- | --- | --- | --- | --- | --- | --- | --- | --- | --- | --- | --- | --- | --- | --- | --- | --- | --- | --- |
|  | 899 | 900 | 915 | 865 | 866 | 867 | 868 | 869 | 881 | 883 | 884 | 885 | 886 | 887 | 888 | 890 | 797 | 799 | 802 | 927 | 929 |  |  |
| 899 |  | 0.845 | 0.459 | 0.307 | 0.205 | 0.213 | 0.179 | 0.176 | 0.155 | 0.460 | 0.464 | 0.443 | 0.371 | 0.265 | 0.109 | 0.135 | 0.302 | 0.277 | 0.330 | 0.026 | 0.036 |  |  |
| 900 | 0.845 |  | 0.700 | 0.527 | 0.428 | 0.427 | 0.394 | 0.375 | 0.088 | 0.573 | 0.530 | 0.550 | 0.486 | 0.352 | 0.240 | 0.281 | 0.422 | 0.383 | 0.431 | 0.194 | 0.221 |  |  |
| 915 | 0.459 | 0.700 |  | 0.487 | 0.382 | 0.383 | 0.334 | 0.310 | -0.088 | 0.411 | 0.366 | 0.388 | 0.423 | 0.299 | 0.276 | 0.309 | 0.280 | 0.251 | 0.370 | 0.415 | 0.406 |  |  |
| 865 | 0.307 | 0.527 | 0.487 |  | 0.947 | 0.952 | 0.887 | 0.876 | 0.056 | 0.319 | 0.217 | 0.301 | 0.474 | 0.556 | 0.480 | 0.592 | 0.640 | 0.641 | 0.647 | 0.434 | 0.494 |  |  |
| 866 | 0.205 | 0.428 | 0.382 | 0.947 |  | 0.988 | 0.977 | 0.959 | 0.048 | 0.241 | 0.176 | 0.223 | 0.384 | 0.563 | 0.446 | 0.583 | 0.588 | 0.606 | 0.590 | 0.431 | 0.512 |  |  |
| 867 | 0.213 | 0.427 | 0.383 | 0.952 | 0.988 |  | 0.972 | 0.962 | 0.048 | 0.248 | 0.172 | 0.236 | 0.414 | 0.569 | 0.450 | 0.571 | 0.601 | 0.617 | 0.596 | 0.424 | 0.504 |  |  |
| 868 | 0.179 | 0.394 | 0.334 | 0.887 | 0.977 | 0.972 |  | 0.988 | 0.029 | 0.211 | 0.162 | 0.200 | 0.358 | 0.571 | 0.426 | 0.561 | 0.554 | 0.568 | 0.536 | 0.423 | 0.506 |  |  |
| 869 | 0.176 | 0.375 | 0.310 | 0.876 | 0.959 | 0.962 | 0.988 |  | 0.012 | 0.191 | 0.137 | 0.187 | 0.371 | 0.590 | 0.418 | 0.538 | 0.569 | 0.582 | 0.538 | 0.394 | 0.467 |  |  |
| 881 | 0.155 | 0.088 | -0.088 | 0.056 | 0.048 | 0.048 | 0.029 | 0.012 |  | 0.197 | 0.269 | 0.183 | -0.019 | 0.023 | 0.117 | 0.154 | 0.129 | 0.131 | -0.019 | 0.034 | 0.052 |  |  |
| 883 | 0.460 | 0.573 | 0.411 | 0.319 | 0.241 | 0.248 | 0.211 | 0.191 | 0.197 |  | 0.908 | 0.946 | 0.600 | 0.317 | 0.204 | 0.167 | 0.459 | 0.408 | 0.415 | 0.169 | 0.174 |  |  |
| 884 | 0.464 | 0.530 | 0.366 | 0.217 | 0.176 | 0.172 | 0.162 | 0.137 | 0.269 | 0.908 |  | 0.848 | 0.460 | 0.265 | 0.127 | 0.126 | 0.329 | 0.296 | 0.317 | 0.148 | 0.156 |  |  |
| 885 | 0.443 | 0.550 | 0.388 | 0.301 | 0.223 | 0.236 | 0.200 | 0.187 | 0.183 | 0.946 | 0.848 |  | 0.652 | 0.371 | 0.205 | 0.160 | 0.426 | 0.367 | 0.379 | 0.142 | 0.158 |  |  |
| 886 | 0.371 | 0.486 | 0.423 | 0.474 | 0.384 | 0.414 | 0.358 | 0.371 | -0.019 | 0.600 | 0.460 | 0.652 |  | 0.710 | 0.571 | 0.364 | 0.524 | 0.436 | 0.455 | 0.116 | 0.136 |  |  |
| 887 | 0.265 | 0.352 | 0.299 | 0.556 | 0.563 | 0.569 | 0.571 | 0.590 | 0.023 | 0.317 | 0.265 | 0.371 | 0.710 |  | 0.643 | 0.584 | 0.475 | 0.452 | 0.397 | 0.347 | 0.346 |  |  |
| 888 | 0.109 | 0.240 | 0.276 | 0.480 | 0.446 | 0.450 | 0.426 | 0.418 | 0.117 | 0.204 | 0.127 | 0.205 | 0.571 | 0.643 |  | 0.842 | 0.399 | 0.303 | 0.218 | 0.350 | 0.366 |  |  |
| 890 | 0.135 | 0.281 | 0.309 | 0.592 | 0.583 | 0.571 | 0.561 | 0.538 | 0.154 | 0.167 | 0.126 | 0.160 | 0.364 | 0.584 | 0.842 |  | 0.414 | 0.369 | 0.281 | 0.488 | 0.514 |  |  |
| 797 | 0.302 | 0.422 | 0.280 | 0.640 | 0.588 | 0.601 | 0.554 | 0.569 | 0.129 | 0.459 | 0.329 | 0.426 | 0.524 | 0.475 | 0.399 | 0.414 |  | 0.957 | 0.710 | 0.253 | 0.297 |  |  |
| 799 | 0.277 | 0.383 | 0.251 | 0.641 | 0.606 | 0.617 | 0.568 | 0.582 | 0.131 | 0.408 | 0.296 | 0.367 | 0.436 | 0.452 | 0.303 | 0.369 | 0.957 |  | 0.768 | 0.261 | 0.310 |  |  |
| 802 | 0.330 | 0.431 | 0.370 | 0.647 | 0.590 | 0.596 | 0.536 | 0.538 | -0.019 | 0.415 | 0.317 | 0.379 | 0.455 | 0.397 | 0.218 | 0.281 | 0.710 | 0.768 |  | 0.209 | 0.253 |  |  |
| 927 | 0.026 | 0.194 | 0.415 | 0.434 | 0.431 | 0.424 | 0.423 | 0.394 | 0.034 | 0.169 | 0.148 | 0.142 | 0.116 | 0.347 | 0.350 | 0.488 | 0.253 | 0.261 | 0.209 |  | 0.961 |  |  |
| 929 | 0.036 | 0.221 | 0.406 | 0.494 | 0.512 | 0.504 | 0.506 | 0.467 | 0.052 | 0.174 | 0.156 | 0.158 | 0.136 | 0.346 | 0.366 | 0.514 | 0.297 | 0.310 | 0.253 | 0.961 |  |  |  |

|  |  |  | |  |  |  |  |  |  |  |  |  |  |  |  |  |  |  |  |  |  |  |  |  |  |  |
| --- | --- | --- | --- | --- | --- | --- | --- | --- | --- | --- | --- | --- | --- | --- | --- | --- | --- | --- | --- | --- | --- | --- | --- | --- | --- | --- |
|  | |  |  | |  |  |  |  |  |  |  |  |  |  |  |  |  |  |  |  |  |  |  |  |  |  |
