# Supplementary material for: NMR Metabolomics Defining Genetic Variation in Pea Seed Metabolites
Source: Front Plant Sci. 2018 Jul 17;9:1022. doi: 10.3389/fpls.2018.01022 (PMC6056766; doi:10.3389/fpls.2018.01022)
Supplement: Supplementary file 5 [file Table_5.docx]

|  |  |  | |  |  |  |  |  |  |  |  |  |  |  |  |  |  |  |  |  |  |  |  |  |  |  |
| --- | --- | --- | --- | --- | --- | --- | --- | --- | --- | --- | --- | --- | --- | --- | --- | --- | --- | --- | --- | --- | --- | --- | --- | --- | --- | --- |
|  | |  |  | |  |  |  |  |  |  |  |  |  |  |  |  |  |  |  |  |  |  |  |  |  |  |

**Supplementary Table S5. Leucine resonances**, **corresponding ppm and bin numbers in the two datasets (Year 1, Year 2)**

| Year 1 | |  |  |  | Year 2 | |  |  |  |  |
| --- | --- | --- | --- | --- | --- | --- | --- | --- | --- | --- |
| bin | Start (ppm) | End (ppm) | Chemical shift (ppm) |  | bin | Start (ppm) | End (ppm) | Chemical shift (ppm) |  | Assignment |
| 900 | 0.996045804 | 0.981517465 | 0.9893 |  |  |  |  |  |  | *Valine doublet methyl 8* |
| 901 | 0.981517465 | 0.972971384 | 0.977 |  | 922 | 0.981517 | 0.971262 | 0.977 |  | Leucine triplet 8,9 |
| 902 | 0.972971384 | 0.961861478 | 0.9653 |  | 923 | 0.971262 | 0.961434 | 0.9653 |  | Leucine triplet 8,9 |
|  |  |  |  |  | 924 | 0.961434 | 0.955879 | 0.9584 |  | *Isoleucine triplet methyl 8* |
|  |  |  |  |  | 925 | 0.955879 | 0.950324 |  |  | *not assigned* |
| 903 | 0.961861478 | 0.948187747 | 0.9494 |  | 926 | 0.950324 | 0.947333 | 0.9494 |  | Leucine triplet 8,9 |
| 904 | 0.948187747 | 0.938787057 | 0.9436 |  | 927 | 0.947333 | 0.940496 | 0.9436 |  | *Isoleucine triplet methyl 8* |
