# Supplementary material for: NMR Metabolomics Defining Genetic Variation in Pea Seed Metabolites
Source: Front Plant Sci. 2018 Jul 17;9:1022. doi: 10.3389/fpls.2018.01022 (PMC6056766; doi:10.3389/fpls.2018.01022)
Supplement: Supplementary file 6 [file Table_6.docx]

### **Supplementary Table S6. Correlations between leucine resonances.** Bin numbers are given in the top row and first column, with location (J, JIC; P, PGRO) and year (y1, Year 1; y2, Year 2).

A: Correlations over the whole map

B: Correlations for LG II

| A | | | 901 y1J | 902 y1J | 903 y1J | 922 y2J | 923 y2J | 926 y2J | 901 y1P | 902 y1P | 903 y1P | 923 y2P | 926 y2P |  |  |
| --- | --- | --- | --- | --- | --- | --- | --- | --- | --- | --- | --- | --- | --- | --- | --- |
| 901 y1J | | |  | 0.840 | 0.574 | 0.107 | 0.135 | 0.175 | 0.266 | 0.238 | 0.253 | 0.244 | 0.195 |  |  |
| 902 y1J | | | 0.840 |  | 0.770 | 0.136 | 0.179 | 0.325 | 0.325 | 0.327 | 0.374 | 0.241 | 0.316 |  | 1 |
| 903 y1J | | | 0.574 | 0.770 |  | 0.079 | 0.136 | 0.379 | 0.277 | 0.290 | 0.451 | 0.172 | 0.344 |  | 0.75 |
| 922 y2J | | | 0.107 | 0.136 | 0.079 |  | 0.947 | 0.529 | 0.286 | 0.383 | 0.365 | 0.243 | 0.174 |  | 0.5 |
| 923 y2J | | | 0.135 | 0.179 | 0.136 | 0.947 |  | 0.579 | 0.365 | 0.480 | 0.462 | 0.321 | 0.243 |  | 0.25 |
| 926 y2J | | | 0.175 | 0.325 | 0.379 | 0.529 | 0.579 |  | 0.199 | 0.308 | 0.496 | 0.212 | 0.514 |  | 0 |
| 901 y1P | | | 0.266 | 0.325 | 0.277 | 0.286 | 0.365 | 0.199 |  | 0.908 | 0.777 | 0.392 | 0.183 |  |  |
| 902 y1P | | | 0.238 | 0.327 | 0.290 | 0.383 | 0.480 | 0.308 | 0.908 |  | 0.851 | 0.479 | 0.268 |  |  |
| 903 y1P | | | 0.253 | 0.374 | 0.451 | 0.365 | 0.462 | 0.496 | 0.777 | 0.851 |  | 0.345 | 0.339 |  |  |
| 923 y2P | | | 0.244 | 0.241 | 0.172 | 0.243 | 0.321 | 0.212 | 0.392 | 0.479 | 0.345 |  | 0.479 |  |  |
| 926 y2P | | | 0.195 | 0.316 | 0.344 | 0.174 | 0.243 | 0.514 | 0.183 | 0.268 | 0.339 | 0.479 |  |  |  |
|  | | |  |  |  |  |  |  |  |  |  |  |  |  |  |
|  |  |  |  |  |  |  |  |  |  |  |  |  |  |  |  |
|  | | |  |  |  |  |  |  |  |  |  |  |  |  |  |
| B | | | 901 y1J | 902 y1J | 903 y1J | 922 y2J | 923 y2J | 926 y2J | 901 y1P | 902 y1P | 903 y1P | 923 y2P | 926 y2P |  |  |
| 901 y1J | | |  | 0.890 | 0.781 | 0.290 | 0.534 | 0.404 | 0.661 | 0.642 | 0.531 | 0.655 | 0.641 |  |  |
| 902 y1J | | | 0.890 |  | 0.879 | 0.326 | 0.531 | 0.476 | 0.695 | 0.694 | 0.607 | 0.665 | 0.677 |  |  |
| 903 y1J | | | 0.781 | 0.879 |  | 0.280 | 0.519 | 0.558 | 0.629 | 0.694 | 0.706 | 0.700 | 0.763 |  |  |
| 922 y2J | | | 0.290 | 0.326 | 0.280 |  | 0.878 | 0.560 | 0.302 | 0.358 | 0.309 | 0.474 | 0.499 |  |  |
| 923 y2J | | | 0.534 | 0.531 | 0.519 | 0.878 |  | 0.595 | 0.511 | 0.614 | 0.524 | 0.740 | 0.703 |  |  |
| 926 y2J | | | 0.404 | 0.476 | 0.558 | 0.560 | 0.595 |  | 0.407 | 0.547 | 0.726 | 0.456 | 0.792 |  |  |
| 901 y1P | | | 0.661 | 0.695 | 0.629 | 0.302 | 0.511 | 0.407 |  | 0.915 | 0.773 | 0.637 | 0.467 |  |  |
| 902 y1P | | | 0.642 | 0.694 | 0.694 | 0.358 | 0.614 | 0.547 | 0.915 |  | 0.911 | 0.796 | 0.659 |  |  |
| 903 y1P | | | 0.531 | 0.607 | 0.706 | 0.309 | 0.524 | 0.726 | 0.773 | 0.911 |  | 0.697 | 0.705 |  |  |
| 923 y2P | | | 0.655 | 0.665 | 0.700 | 0.474 | 0.740 | 0.456 | 0.637 | 0.796 | 0.697 |  | 0.768 |  |  |
| 926 y2P | | | 0.641 | 0.677 | 0.763 | 0.499 | 0.703 | 0.792 | 0.467 | 0.659 | 0.705 | 0.768 |  |  |  |
