# Supplementary material for: NMR Metabolomics Defining Genetic Variation in Pea Seed Metabolites
Source: Front Plant Sci. 2018 Jul 17;9:1022. doi: 10.3389/fpls.2018.01022 (PMC6056766; doi:10.3389/fpls.2018.01022)
Supplement: Supplementary file 7 [file Table_7.docx]

### **Supplementary Table S7. Summary of compounds assigned to NMR bins, together with their chemical shifts (ppm)**

| Chemical shift, Year 1 | Assignment | Chemical shift, Year 2 | Assignment |
| --- | --- | --- | --- |
|  |  | 1.4785 | Alanine doublet methyl 6 |
|  |  | 1.493 | Alanine doublet methyl 6 |
| 3.7805 | Alanine/Arginine or glutamate doublet of doublets |  |  |
|  |  | 1.33618 | aliphatic tentative: Hesperidin or Delphinidin |
|  |  |  |  |
| 5.494276 | anomeric or alkene, unknown | 5.494276 | anomeric or alkene, unknown |
| 5.49043 | anomeric or alkene, unknown (tentative: Dodecenic acid) |  |  |
|  |  | 1.794 | Arginine multiplet 6 |
|  |  | 1.6061 | Arginine multiplet 7 |
| 3.2469 | Arginine triplet 8 |  |  |
|  |  | 6.158306 | aromatic or amide, unknown |
|  |  | 6.147196 | aromatic or amide, unknown |
| 8.59864 | aromatic or amide, unknown |  |  |
| 7.359458 | aromatic, tentative: Naringin | 7.359458 | aromatic, tentative: naringin |
| 6.966766 | aromatic, tentative: p-Coumarate | 6.966766 | aromatic, tentative: p-Coumarate |
| 6.983003 | aromatic, tentative: Rutin | 6.983003 | aromatic, tentative: Rutin |
| 6.371958 | aromatic, unknown | 6.371958 | aromatic, unknown |
| 6.67449 | aromatic, unknown | 6.67449 | aromatic, unknown |
| 7.05009 | aromatic, unknown | 7.05009 | aromatic, unknown |
| 7.084702 | aromatic, unknown | 7.084702 | aromatic, unknown |
| 7.085984 | aromatic, unknown |  |  |
|  |  | 7.091111 | aromatic, unknown |
| 7.13726 | aromatic, unknown | 7.13726 | aromatic, unknown |
| 7.183409 | aromatic, unknown | 7.183409 | aromatic, unknown |
|  |  | 7.237249 | aromatic, unknown |
|  |  | 7.489359 | aromatic, unknown |
| 7.591484 | aromatic, unknown | 7.591484 | aromatic, unknown |
| 6.298462 | aromatic, unknown (Chlorogenic acid) |  |  |
| 7.026161 | aromatic, unknown (Chlorogenic acid) | 7.026161 | aromatic, unknown (Chlorogenic acid) |
| 7.031716 | aromatic, unknown (tentative: Chlorogenic acid) | 7.031716 | aromatic, unknown (tentative: Chlorogenic acid) |
| 7.686773 | aromatic, unknown (Folic acid) | 7.686773 | aromatic, unknown (Folic acid) |
| 7.224003 | aromatic, unknown (tentative: tyramine) | 7.224003 | aromatic, unknown (tentative: tyramine) |
| 2.7039 | Aspartic acid doublet of doublets 6 |  |  |
| 2.801 | Aspartic acid doublet of doublets 6 |  |  |
| 2.836 | Aspartic acid doublet of doublets 6 |  |  |
| 5.5804 | doublet satellite Sucrose 7 |  |  |
|  |  | 1.9226 | GABA multiplet 5 |
| 2.2853 | GABA triplet 4 | 2.3002 | GABA triplet 4 |
| 2.3002 | GABA triplet 4 | 2.2853 | GABA triplet 4 |
| 3.0119 | GABA triplet 6 |  |  |
|  |  |  |  |
| 2.1603 | Glutamate multiplet 6/glutamine multiplet 6 | 2.1147 | Glutamate multiplet 6/glutamine multiplet 6 |
|  |  | 2.1245 | Glutamate multiplet 6/glutamine multiplet 6 |
|  |  | 2.1305 | Glutamate multiplet 6/glutamine multiplet 6 |
|  |  | 2.1403 | Glutamate multiplet 6/glutamine multiplet 6 |
|  |  | 2.1446 | Glutamate multiplet 6/glutamine multiplet 6 |
|  |  | 2.1543 | Glutamate multiplet 6/glutamine multiplet 6 |
|  |  | 2.1603 | Glutamate multiplet 6/glutamine multiplet 6 |
|  |  | 2.3396 | Glutamate multiplet 7 |
|  |  | 2.348 | Glutamate multiplet 7 |
|  |  | 2.3545 | Glutamate multiplet 7 |
| 2.3643 | Glutamate multiplet 7 |  |  |
|  |  | 2.3712 | Glutamate multiplet 7 |
| 2.4081 | Glutamine multiplet 7 |  |  |
| 2.4942 | Glutamine m 7 |  |  |
|  |  |  |  |
|  |  | 2.1761 | Glutamate/Glutamine |
|  |  | 2.1918 | Glutamate/Glutamine |
|  |  | 2.1676 | Glutamate/Glutamine/Glutathione |
| 2.1957 | Glutathione | 2.1957 | Glutathione |
|  |  | 2.584 | Glutathione red form multiplet 9 |
|  |  | 2.9718 | Glutathione reduced doublet of doublets 2 |
| 3.0028 | Glutathione doublet of doublets 2 |  |  |
| 4.5726 | Glutathione reduced doublet of doublets 3 |  |  |
| 4.5863 | Glutathione reduced doublet of doublets 3 | 4.5863 | Glutathione reduced doublet of doublets 3 |
| 2.9141 | Glutathione reduced form | 2.9141 | Glutathione reduced form |
| 7.106494 | Hesperidin | 7.106494 | Hesperidin |
| 7.122304 | Hesperidin | 7.122304 | Hesperidin |
|  |  | 8.365332 | hetero-aromatic unknown |
| 8.542663 | hetero-aromatic, unknown |  |  |
| 2.0388 | homoserine multiplet 3 or glutamate multiplet 6 | 2.0388 | homoserine multiplet 3 or glutamate multiplet 6 |
| 2.0415 | homoserine multiplet 3 or glutamate multiplet 6 |  |  |
| 2.0247 | Homoserine multiplet 7 | 2.0247 | Homoserine multiplet 7 |
|  |  | 1.1385 | Isoleucine doublet 9 |
|  |  | 1.1525 | Isoleucine doublet 9 |
|  |  | 1.0078 | Isoleucine doublet methyl 9 |
| 1.0219 | Isoleucine doublet methyl 9 | 1.0219 | Isoleucine doublet methyl 9 |
|  |  | 1.2265 | Isoleucine multiplet 7 |
|  |  | 1.2407 | Isoleucine multiplet 7 |
|  |  | 1.2784 | Isoleucine multiplet 7 |
|  |  | 1.2932 | Isoleucine multiplet 7 |
| 1.435 | Isoleucine multiplet 7 | 1.435 | Isoleucine multiplet 7 |
| 1.4441 | Isoleucine multiplet 7 | 1.4441 | Isoleucine multiplet 7 |
| 1.4511 | Isoleucine multiplet 7 | 1.4511 | Isoleucine multiplet 7 |
| 1.4603 | Isoleucine multiplet 7 | 1.4603 | Isoleucine multiplet 7 |
|  |  | 1.4652 | Isoleucine multiplet 7 |
|  |  | 1.9717 | Isoleucine multiplet 9 |
| 2.0035 | Isoleucine multiplet 9 |  |  |
| 0.9287 | Isoleucine triplet methyl 8 | 0.9287 | Isoleucine triplet methyl 8 |
| 0.9436 | Isoleucine triplet methyl 8 | 0.9436 | Isoleucine triplet methyl 8 |
|  |  | 0.9584 | Isoleucine triplet methyl 8 |
| 0.9494 | Leucine triplet 8,9 | 0.9494 | Leucine triplet 8,9 |
| 0.9653 | Leucine triplet 8,9 | 0.9653 | Leucine triplet 8,9 |
|  |  | 0.977 | Leucine triplet 8,9 |
| 4.4456 | likely Trigonelline | 4.4456 | likely Trigonelline |
| 3.6461 | Myoinositol triplet 1,3 | 3.6461 | Myoinositol triplet 1,3 |
|  |  | 3.2862 | Myoinositol triplet 2 |
| 3.5336 | Myoinositol doublet of doublets 6,4 + Glucose | 3.5336 | MyoInositol doublet of doublets 6,4 + Glucose |
|  |  | 3.5272 | MyoInositol doublet of doublets 6,4 + Glucose |
| 3.6073 | MyoInositol triplet 1,3 | 3.6073 | MyoInositol triplet 1,3 |
| 7.3406 | Phenylalanine |  |  |
| 7.3679 | Phenylalanine | 7.3679 | Phenylalanine |
|  |  | 7.370995 | phenylalanine |
| 7.3735 | Phenylalanine |  |  |
| 7.3844 | Phenylalanine | 7.3844 | Phenylalanine |
| 7.3943 | Phenylalanine | 7.3943 | Phenylalanine |
| 7.4217 | Phenylalanine |  |  |
| 7.4334 | Phenylalanine | 7.4439 | Phenylalanine |
|  |  | 3.5925 | Raffinose multiplet 10,12 |
| 4.0873 | Raffinose m 19, 9, 4 /Stachyose m 24, 19, 35, 4 |  |  |
| 3.9501 | Raffinose triplet, 23 |  |  |
| 3.9636 | Raffinose triplet, 23 |  |  |
| 3.9757 | Raffinose triplet, 23 |  |  |
| 3.9969 | Stachyose multiplet, 26, 9 |  |  |
|  |  | 3.664132 | Sucrose, Raffinose, Verbascose or Stachyose |
| 5.491285 | tentative: Methyl maleic acid |  |  |
|  |  | 7.318437 | tentative: Phenylacetic acid |
| 8.1045 | Trigonelline triplet 4 | 8.1045 | Trigonelline triplet 4 |
|  |  | 8.8377 | Trigonelline multiplet 3 and 5 |
|  |  | 8.8524 | Trigonelline multiplet 3 and 5 |
|  |  | 9.1329 | Trigonelline singlet , 1 |
|  |  | 8.0761 | Trigonelline triplet 4 |
|  |  | 8.0903 | Trigonelline triplet 4 |
| 7.195801 | Tyrosine |  |  |
| 7.21973 | Tyrosine |  |  |
| 3.0881 | Tyrosine doublet of doublets 8 |  |  |
| 6.8945 | Tyrosine multiplet 2,6 |  |  |
| 6.9214 | Tyrosine multiplet 2,6 |  |  |
|  |  | 7.1898 | Tyrosine multiplet 3,5 |
| 7.2029 | Tyrosine multiplet 3,5 | 7.2029 | Tyrosine multiplet 3,5 |
| 7.2127 | Tyrosine multiplet 3,5 | 7.2127 | Tyrosine multiplet 3,5 |
|  |  | 0.9893 | Valine doublet methyl 8 |
|  |  | 1.0033 | Valine doublet methyl 8 |
|  |  | 1.0397 | Valine methyl 7 |
| 1.0538 | Valine methyl 7 | 1.0538 | Valine methyl 7 |
| 4.1924 | Verbascose |  |  |
|  |  | 4.202 | Verbascose |
|  |  | 4.2077 | Verbascose |
| 4.402514 | Verbascose |  |  |
|  |  | 4.0373 | Verbascose / Raffinose (raffinose m, 19,9,4) |
| 4.24783 | Verbascose, Stachyose and Threonine |  |  |
|  |  | 5.0054 | Verbascose/Raffinose |
| 4.0023 | Verbascose/Stachyose | 4.0023 | Verbascose/Stachyose |
| 4.9978 | Verbascose/Stachyose/Raffinose | 4.9978 | Verbascose/Stachyose/Raffinose |
| 5.514359 | vinylic, tentative: 5-dodecenoic acid |  |  |
|  |  |  |  |
| Below are the signals for which no compound was assigned | | | |
| 0.9092 | unknown | 0.9092 | unknown |
| 0.9361 | unknown | 0.9361 | unknown |
|  |  | 1.1847 | unknown |
|  |  | 1.1974 | unknown |
|  |  | 1.2097 | unknown |
|  |  | 1.2343 | unknown |
| 1.3993 | unknown |  |  |
| 1.4132 | unknown |  |  |
|  |  | 1.7804 | unknown |
|  |  | 1.8127 | unknown |
|  |  | 1.8278 | unknown |
|  |  | 1.8422 | unknown |
|  |  | 1.8568 | unknown |
|  |  | 1.8663 | unknown |
|  |  | 1.9866 | unknown |
|  |  | 1.9916 | unknown |
| 2.0306 | unknown | 2.0306 | unknown |
|  |  | 2.0681 | unknown |
|  |  | 2.082 | unknown |
|  |  | 2.2744 | unknown |
|  |  | 2.3864 | unknown |
|  |  | 2.5267 | unknown |
|  |  | 2.5572 | unknown |
| 2.6227 | unknown |  |  |
|  |  | 2.6618 | unknown |
| 2.6871 | unknown |  |  |
|  |  | 2.6925 | unknown |
| 2.8553 | unknown |  |  |
| 2.87 | unknown |  |  |
|  |  | 3.0204 | unknown |
| 3.0817 | unknown |  |  |
|  |  | 3.2087 | unknown |
|  |  | 3.2259 | unknown |
|  |  | 3.2346 | unknown |
| 3.3192 | unknown |  |  |
| 3.3466 | unknown |  |  |
| 3.3654 | unknown |  |  |
| 3.5177 | unknown | 3.5177 | unknown |
| 3.6127 | unknown |  |  |
| 3.6175 | unknown |  |  |
| 3.6218 | unknown | 3.6218 | unknown |
| 3.6581 | unknown |  |  |
| 3.7203 | unknown | 3.7203 | unknown |
| 3.7295 | unknown |  |  |
| 3.8471 | unknown | 3.7295 | unknown |
| 3.8602 | unknown |  |  |
| 3.9183 | unknown |  |  |
| 3.9283 | unknown |  |  |
| 3.946 | unknown | 3.946 | unknown |
| 3.9609 | unknown |  |  |
|  |  | 3.986 | unknown |
| 4.0206 | unknown |  |  |
| 4.0273 | unknown | 4.0273 | unknown |
| 4.137 | unknown |  |  |
| 4.1511 | unknown | 4.1511 | unknown |
| 4.1568 | unknown | 4.1568 | unknown |
| 4.1662 | unknown |  |  |
| 4.2886 | unknown |  |  |
| 4.2948 | unknown |  |  |
| 4.3089 | unknown | 4.3089 | unknown |
|  |  | 4.3151 | unknown |
| 4.3592 | unknown |  |  |
| 4.417 | unknown |  |  |
| 4.5101 | unknown |  |  |
| 4.602 | unknown |  |  |
|  |  | 4.8011 | unknown |
| 5.1498 | unknown | 5.1498 | unknown |
| 5.1576 | unknown | 5.1576 | unknown |
| 5.27 | unknown | 5.27 | unknown |
| 5.8997 | unknown |  |  |
| 5.9159 | unknown |  |  |
| 5.9214 | unknown |  |  |
| 5.9305 | unknown |  |  |
| 6.6013 | unknown | 6.6013 | unknown |
|  |  | 6.9415 | unknown |
| 6.9587 | unknown | 6.9587 | unknown |
