# Supplementary material for: NMR Metabolomics Defining Genetic Variation in Pea Seed Metabolites
Source: Front Plant Sci. 2018 Jul 17;9:1022. doi: 10.3389/fpls.2018.01022 (PMC6056766; doi:10.3389/fpls.2018.01022)
Supplement: Supplementary file 8 [file Presentation_1.ZIP › Supplementary Figure S1.pptx]

## Slide 1
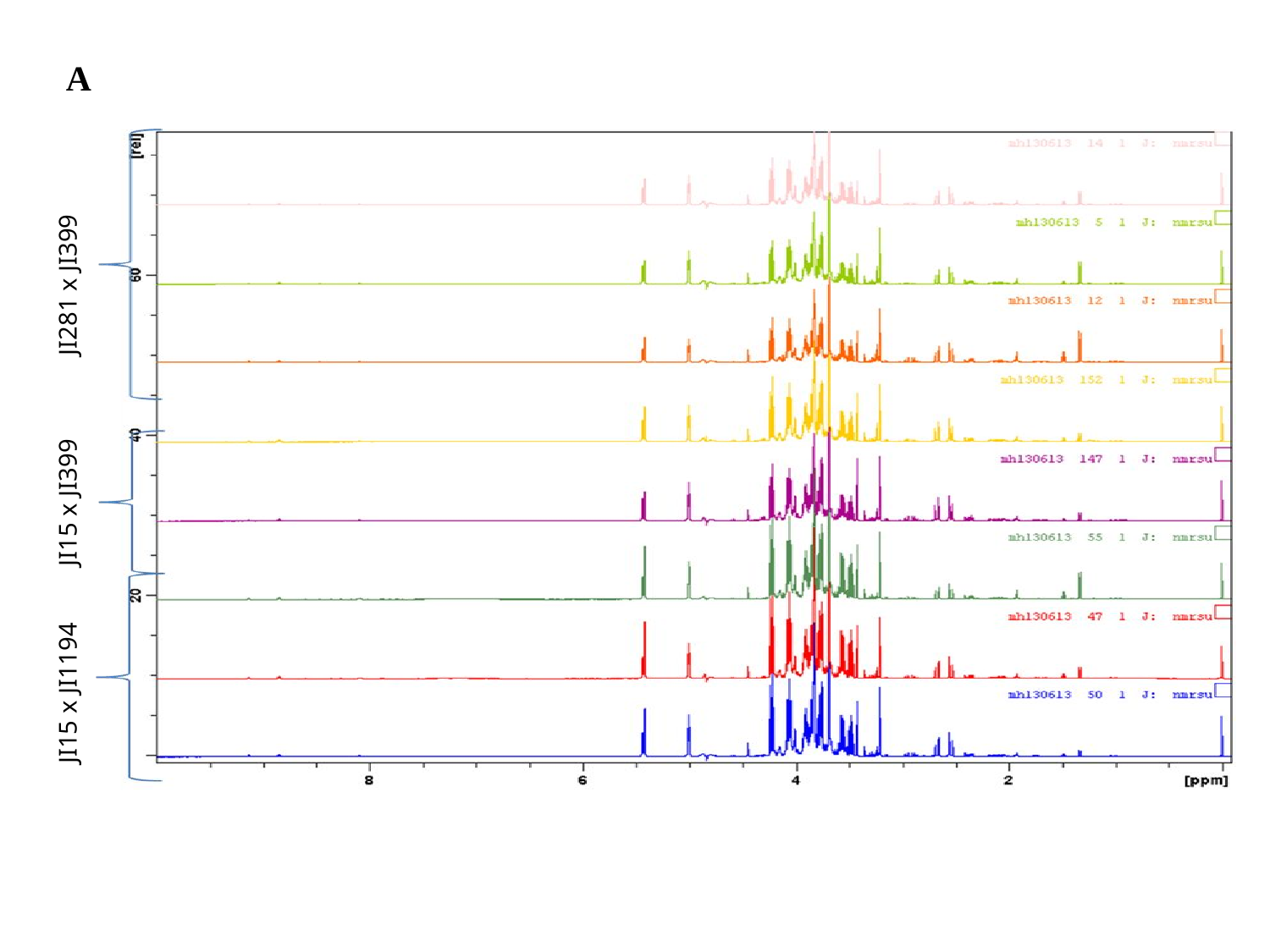

A
JI281 x JI399
JI15 x JI399
JI15 x JI1194

## Slide 2
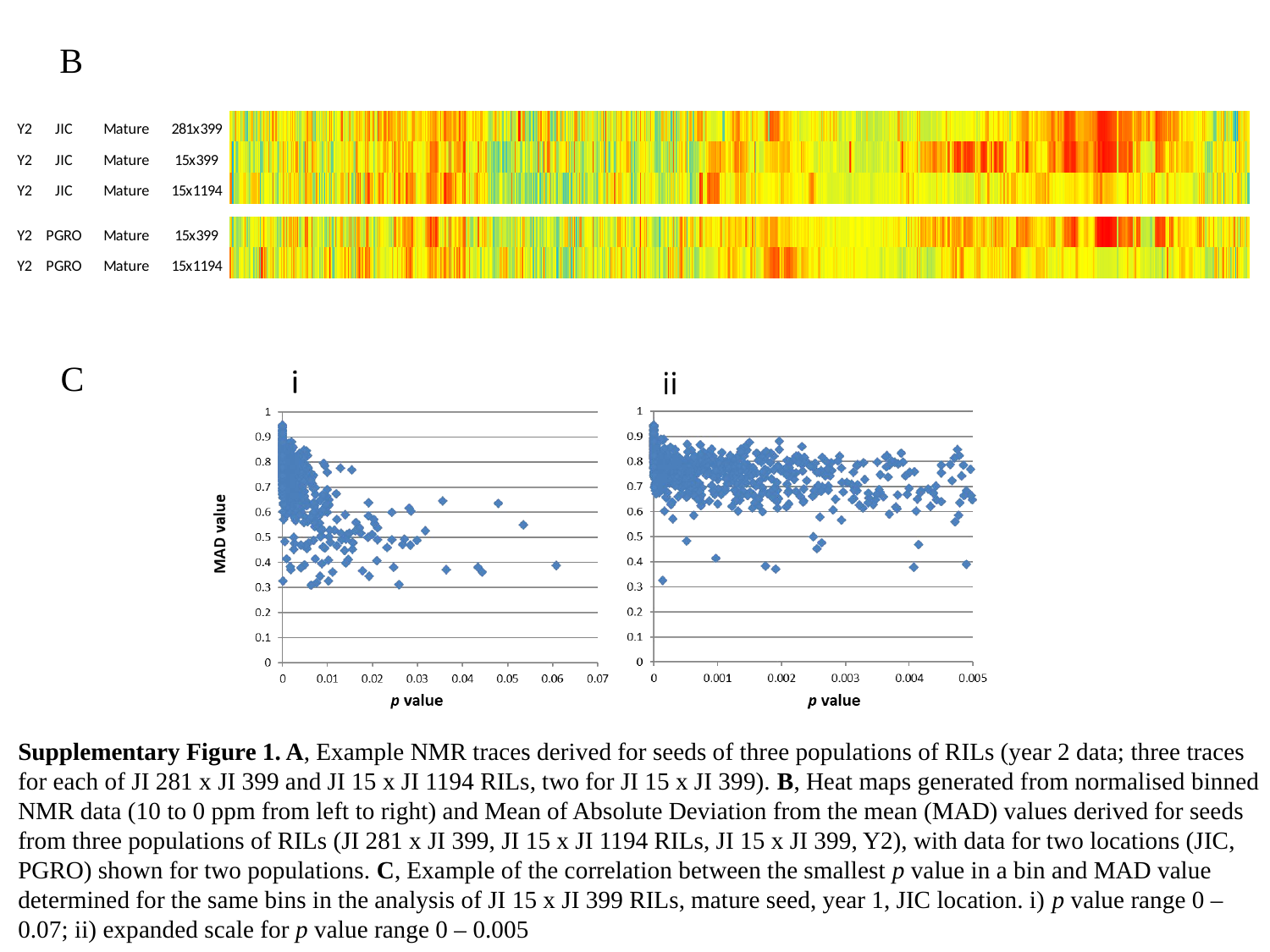

B
C
Supplementary Figure 1. A, Example NMR traces derived for seeds of three populations of RILs (year 2 data; three traces for each of JI 281 x JI 399 and JI 15 x JI 1194 RILs, two for JI 15 x JI 399). B, Heat maps generated from normalised binned NMR data (10 to 0 ppm from left to right) and Mean of Absolute Deviation from the mean (MAD) values derived for seeds from three populations of RILs (JI 281 x JI 399, JI 15 x JI 1194 RILs, JI 15 x JI 399, Y2), with data for two locations (JIC, PGRO) shown for two populations. C, Example of the correlation between the smallest p value in a bin and MAD value determined for the same bins in the analysis of JI 15 x JI 399 RILs, mature seed, year 1, JIC location. i) p value range 0 – 0.07; ii) expanded scale for p value range 0 – 0.005
