# Supplementary material for: NMR Metabolomics Defining Genetic Variation in Pea Seed Metabolites
Source: Front Plant Sci. 2018 Jul 17;9:1022. doi: 10.3389/fpls.2018.01022 (PMC6056766; doi:10.3389/fpls.2018.01022)
Supplement: Supplementary file 8 [file Presentation_1.ZIP › Supplementary Figure S2.docx]

**Supplementary Figure S2**


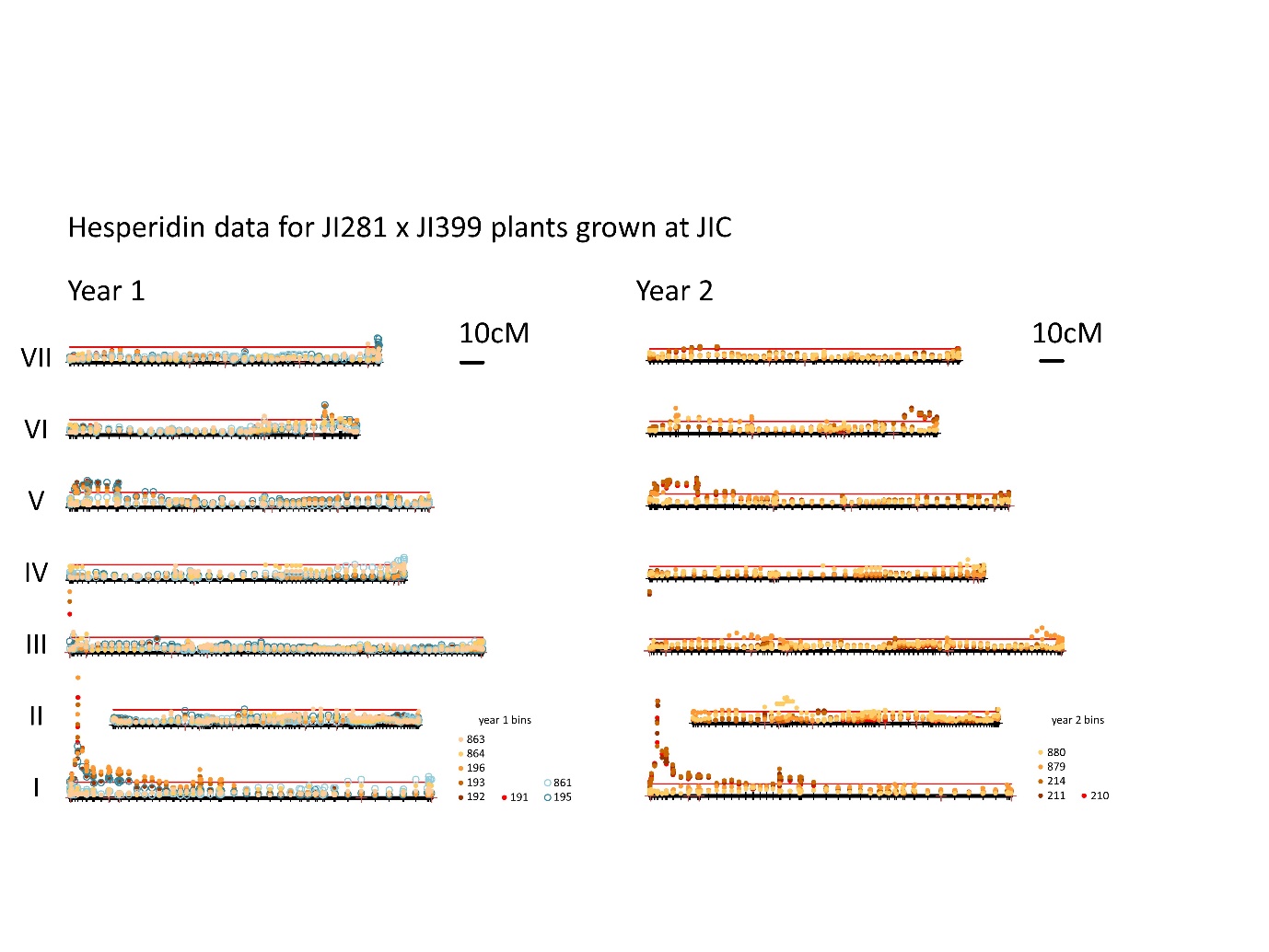


**Figure S2. Mapping variation in hesperidin in JI 281 x JI 399.** The *p* values for hesperidin-related bins are plotted as -log_10_ (*p*) for each marker of the JI 281 x JI 399 population from NMR data collected for seeds of plants grown at JIC in year 1 and year 2. The red line corresponds to the critical *p* value calculated as described in the text. Linkage groups (I – VII) are identified by roman numerals. LG II is offset so that it does not overlap with the peak in LG I. The bins represented by tan coloured dots are bins that contain resonances corresponding to a signal from hesperidin. The red coloured dots (bin 191 in year 1 and bin 210 in year 2) correspond to an unidentified resonance which was the most significant peak at the top of LG I in year 2. The open dots in blue correspond to year 1 bins that overlap with the year 2 bins 214 and 879. In the year 1 plot the bins 861 and 863 do not follow the pattern of the other peaks. There are additional significant *p* values at other locations on the genetic map, including LG groups V, VI and VII that show consistency between years. The peak at the left-hand side of LG III is likely to be an artefact due to the biased selection for the *rb* allele in the samples analysed, as discussed in the text. Scale bar, 10 centimorgans
