# Supplementary material for: NMR Metabolomics Defining Genetic Variation in Pea Seed Metabolites
Source: Front Plant Sci. 2018 Jul 17;9:1022. doi: 10.3389/fpls.2018.01022 (PMC6056766; doi:10.3389/fpls.2018.01022)
Supplement: Supplementary file 8 [file Presentation_1.ZIP › Supplementary Figure S3.docx]

**Supplementary Figure S3**


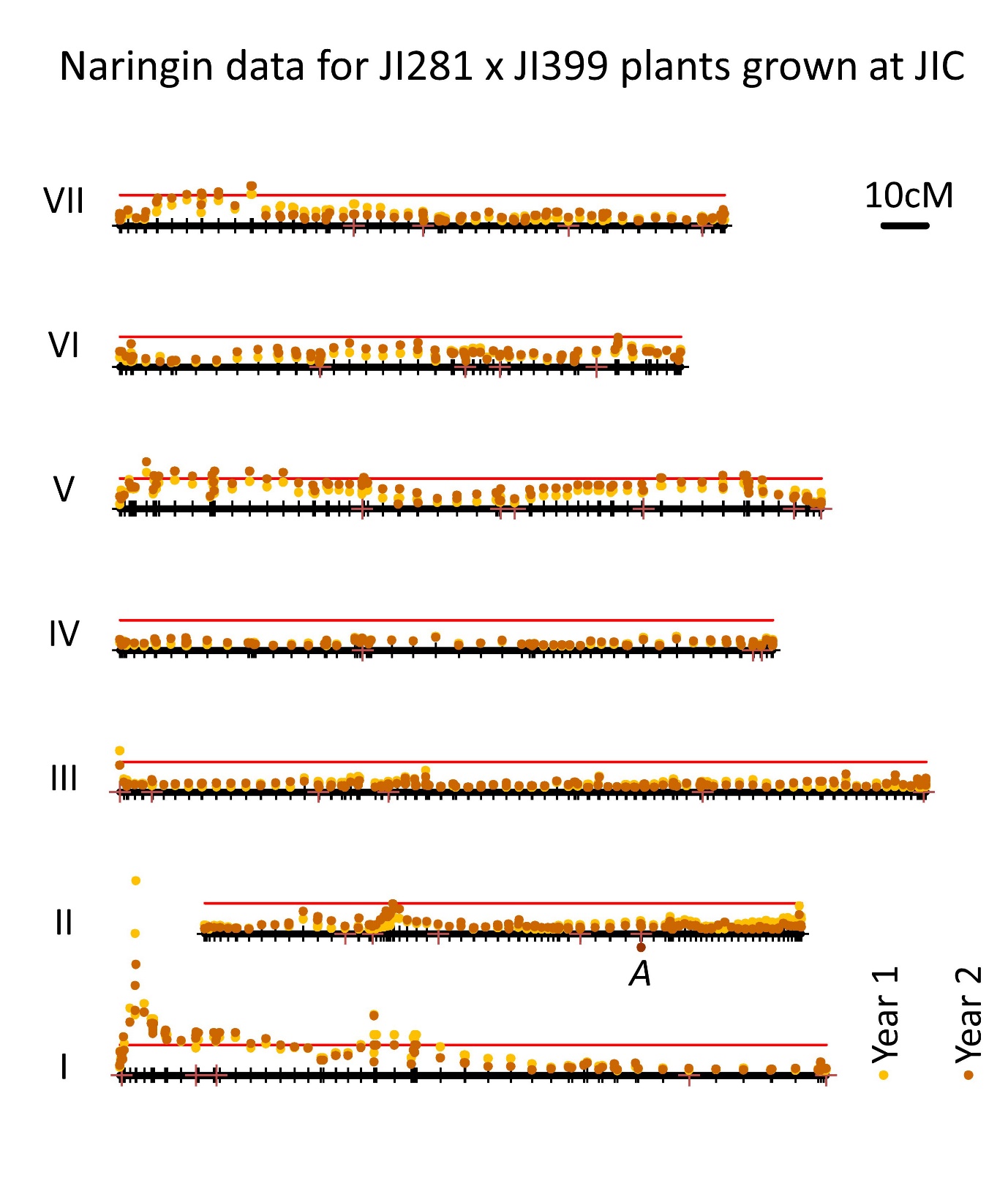


**Figure S3. Mapping variation in naringin in JI 281 x JI 399.** The -log_10_ (*p)* values for the NMR bin that includes the resonance at 7.359458 ppm for year 1 (bin 166) and year 2 (bin 179) are plotted against the pea genetic map of the JI 281 x JI 399 population grown at JIC. This resonance was tentatively assigned to naringin. The red line corresponds to the critical *p* value calculated as described in the text. Linkage groups (I – VII) are identified by roman numerals. LG II (with *A* locus marked) is offset so that it does not overlap with the peak in group I. Scale bar, 10 centimorgans
