# Supplementary material for: NMR Metabolomics Defining Genetic Variation in Pea Seed Metabolites
Source: Front Plant Sci. 2018 Jul 17;9:1022. doi: 10.3389/fpls.2018.01022 (PMC6056766; doi:10.3389/fpls.2018.01022)
Supplement: Supplementary file 8 [file Presentation_1.ZIP › Supplementary Figure S4.docx]

**Supplementary Figure S4**


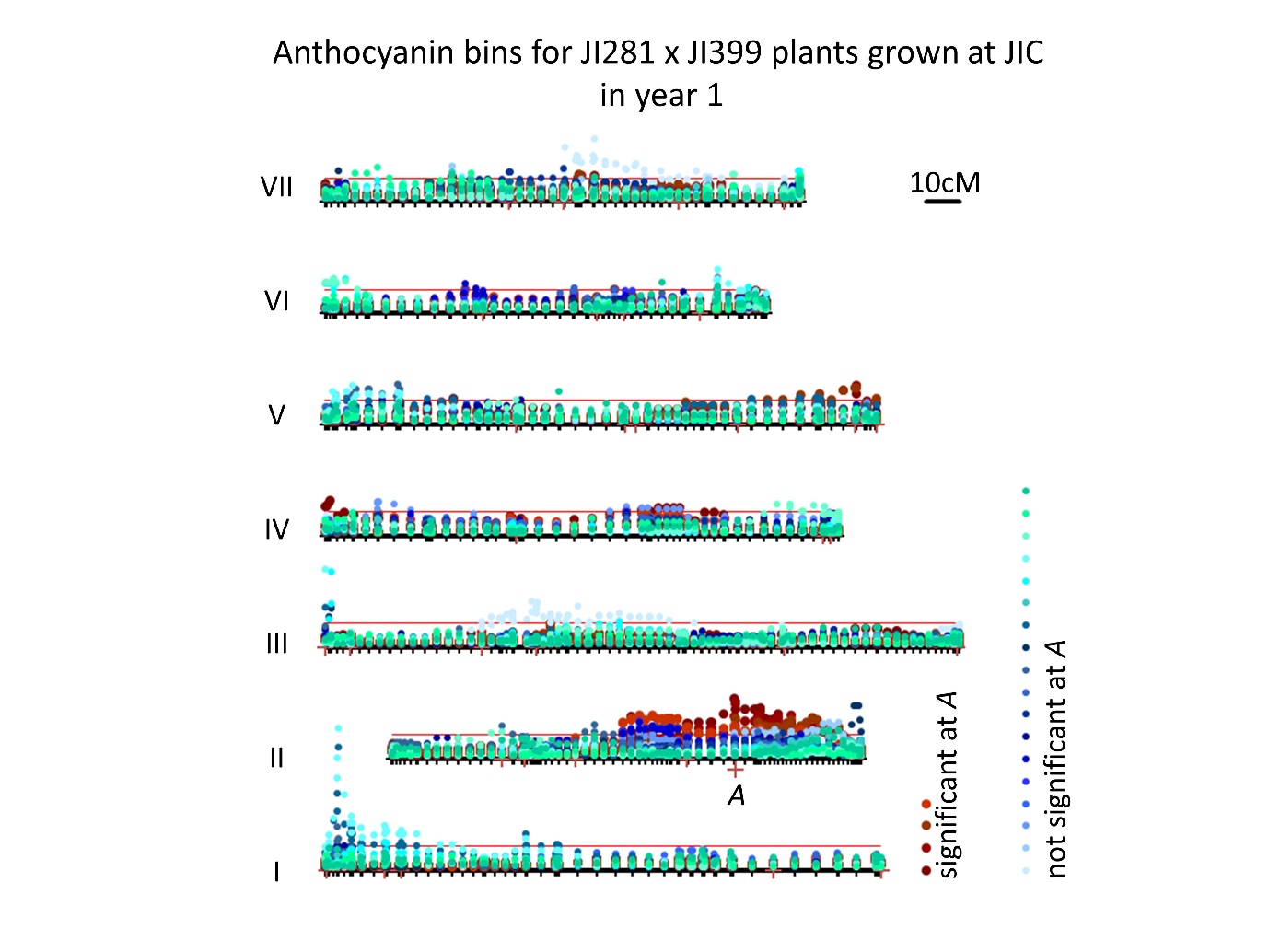


**Figure S4.** **Mapping variation in anthocyanin bins in JI 281 x JI 399.** NMR resonances were assigned to a variety of anthocyanin compounds in the study of Kirby et al. (2013). A small subset of these, corresponding to aglycones, were aligned with the bins analysed in the present study. The correspondences and bins identified are indicated in Supplementary Table 1. A subset of these bins shows statistically significant variation in signal intensity associated with the allele at the *A* locus, labelled on LG II. Data for JIC location (year 1) are shown. Linkage groups (I – VII) are identified by roman numerals. Scale bar, 10 centimorgans
