# Supplementary material for: NMR Metabolomics Defining Genetic Variation in Pea Seed Metabolites
Source: Front Plant Sci. 2018 Jul 17;9:1022. doi: 10.3389/fpls.2018.01022 (PMC6056766; doi:10.3389/fpls.2018.01022)
Supplement: Supplementary file 8 [file Presentation_1.ZIP › Supplementary Figure S5.docx]

### **Supplementary Figure S5**


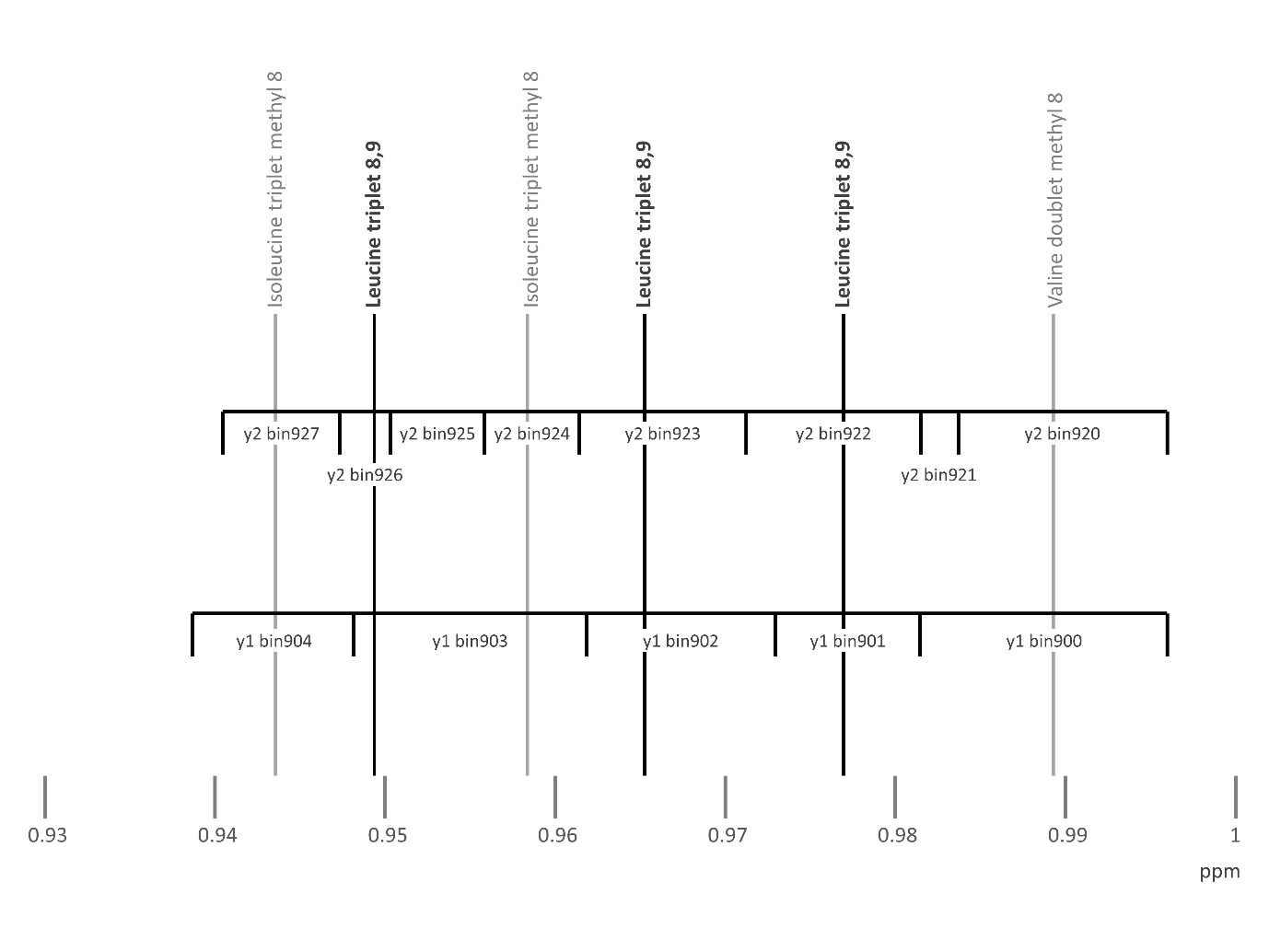


**Figure S5. The correspondence of leucine resonances and NMR bin numbers in the two datasets analysed (Year 1, Year 2).** Leucine resonances are given in black font; additional resonances attributed to other compounds are shown in grey font. The NMR spectrum ppm values are indicated beneath the year 1 bin numbers.
