# Supplementary material for: NMR Metabolomics Defining Genetic Variation in Pea Seed Metabolites
Source: Front Plant Sci. 2018 Jul 17;9:1022. doi: 10.3389/fpls.2018.01022 (PMC6056766; doi:10.3389/fpls.2018.01022)
Supplement: Supplementary file 8 [file Presentation_1.ZIP › Supplementary Figure S6.docx]

### **Supplementary Figure 6**


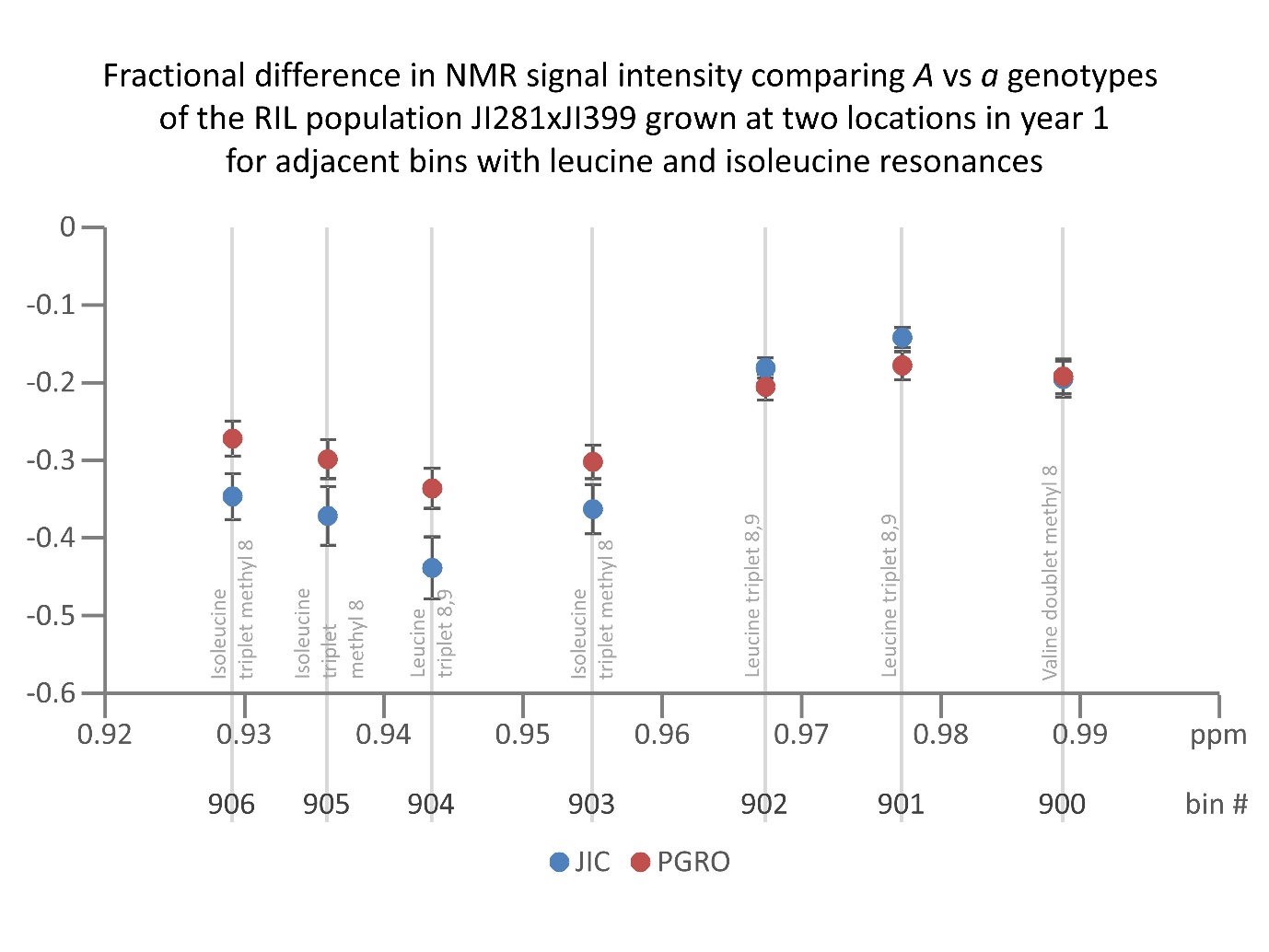


**Figure S6. The direction of the quantitative effect associated with *A* or *a* alleles.** The plot shows the fractional difference in NMR signal intensity between the genotypes *A* and *a* (JI 281 x JI 399 population, two locations, year 1) for adjacent bins with leucine and isoleucine resonances. The consecutive year 1 bins 901 to 906 were associated with leucine or isoleucine and all showed a significant difference between the two alleles (*A,* *a*). The resonance intensities for these bins were compared and the difference in resonance intensity (allele *A* minus allele *a*) divided by the average of these intensities is plotted against the NMR spectrum (ppm).
