# Supplementary material for: NMR Metabolomics Defining Genetic Variation in Pea Seed Metabolites
Source: Front Plant Sci. 2018 Jul 17;9:1022. doi: 10.3389/fpls.2018.01022 (PMC6056766; doi:10.3389/fpls.2018.01022)
Supplement: Supplementary file 9 [file Data_Sheet_1.ZIP › Supplementary File S1 (Genetic mapping).docx]

# Supplementary File S1 (supported by five Supplementary Files S1-Figures M1–M9.xlsx as the genetic mapping data files, which generated the supplementary file mapping figures M1-M9 below)

**Pea recombinant inbred populations and maps**

The recombinant inbred populations used in this study: JI 281 x JI 399, JI 15 x JI 399 and JI 15 x JI 1194 have been described with respect to genetic and cytogenetics by Hall et al. (1997a, b), further described in Ellis et al. (1998), Lacou et al. (1998) and Ellis and Poyser (2002) and discussed with respect to other legume genomes in Choi et al. (2004) and Kalό et al. (2004). Here we have extended the marker density in these populations, notably with the inclusion of sequence defined markers to facilitate alignment with other genomes, and have used the Thread mapper (Cheema et al. 2010) approach to generate these maps. The Sequence Specific Amplified Polymorphism (SSAP) markers (Ellis et al. 1998, Knox et al. 2009) have, because of their high level of presence/absence polymorphism, proved useful for the integration of pea genetic maps where one parent is in common. These maps are described briefly below and data sets are available in accompanying excel files.

The lines JI 15 and JI 399 are each a parent in two populations. This means that the alleles from these crosses segregate in two populations, so the three populations can be analysed together. Accordingly, a map can be drawn for the combined populations, and this facilitates comparison between the different maps of individual populations. This is shown below:

#### **Figure M1. The combined genetic map**


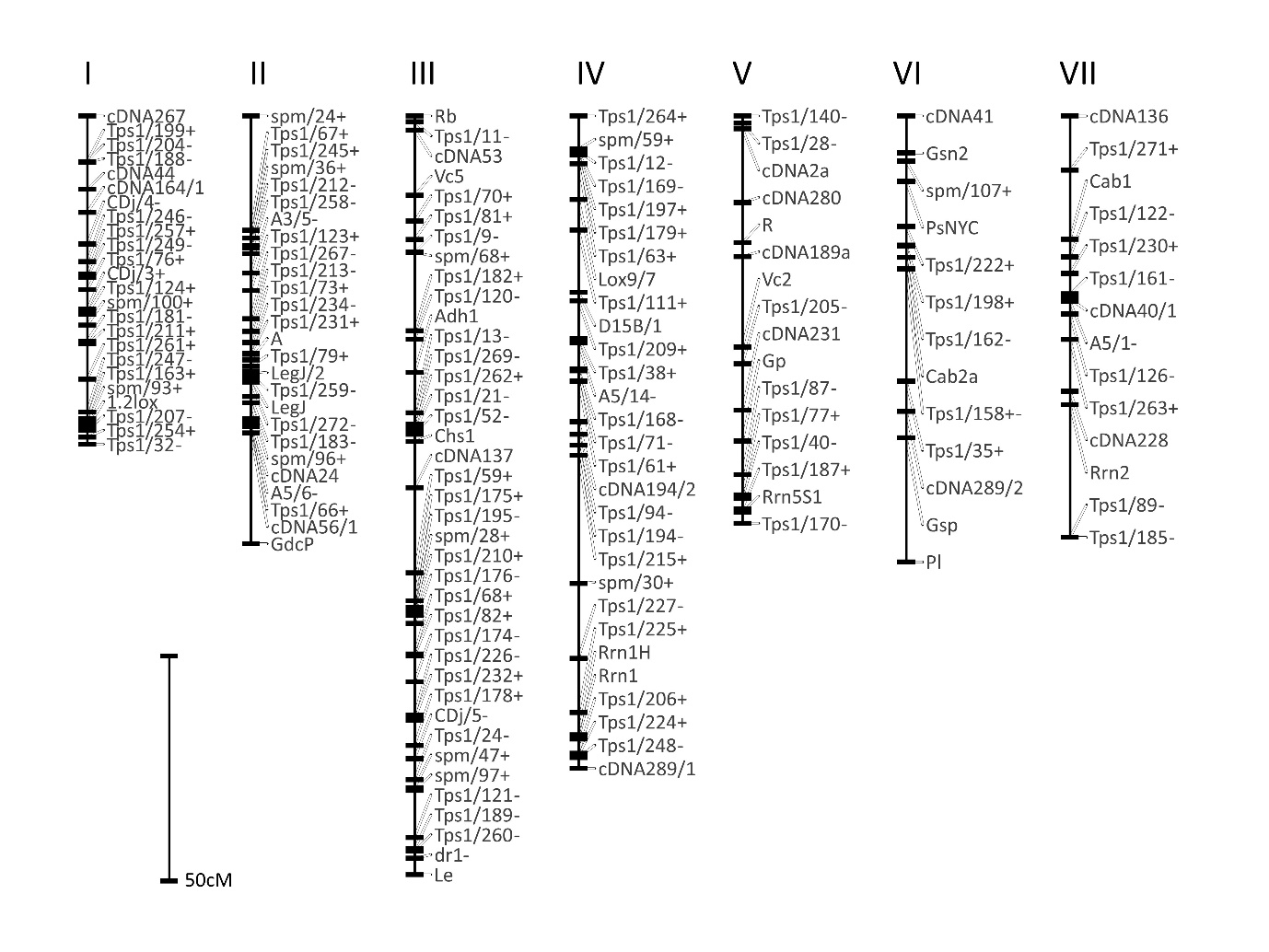


**Figure M1.** A combined genetic map of the three recombinant inbred populations used in this study. The data are available in the excel files **Figures M1 and M2.xlsx**. For this map, marker order and inter-marker distances were calculated using JoinMap version 3.0.

Marker segregation ratios are illustrated in Figure M2.

#### **Figure M2. Segregation ratios in the combined genetic map**


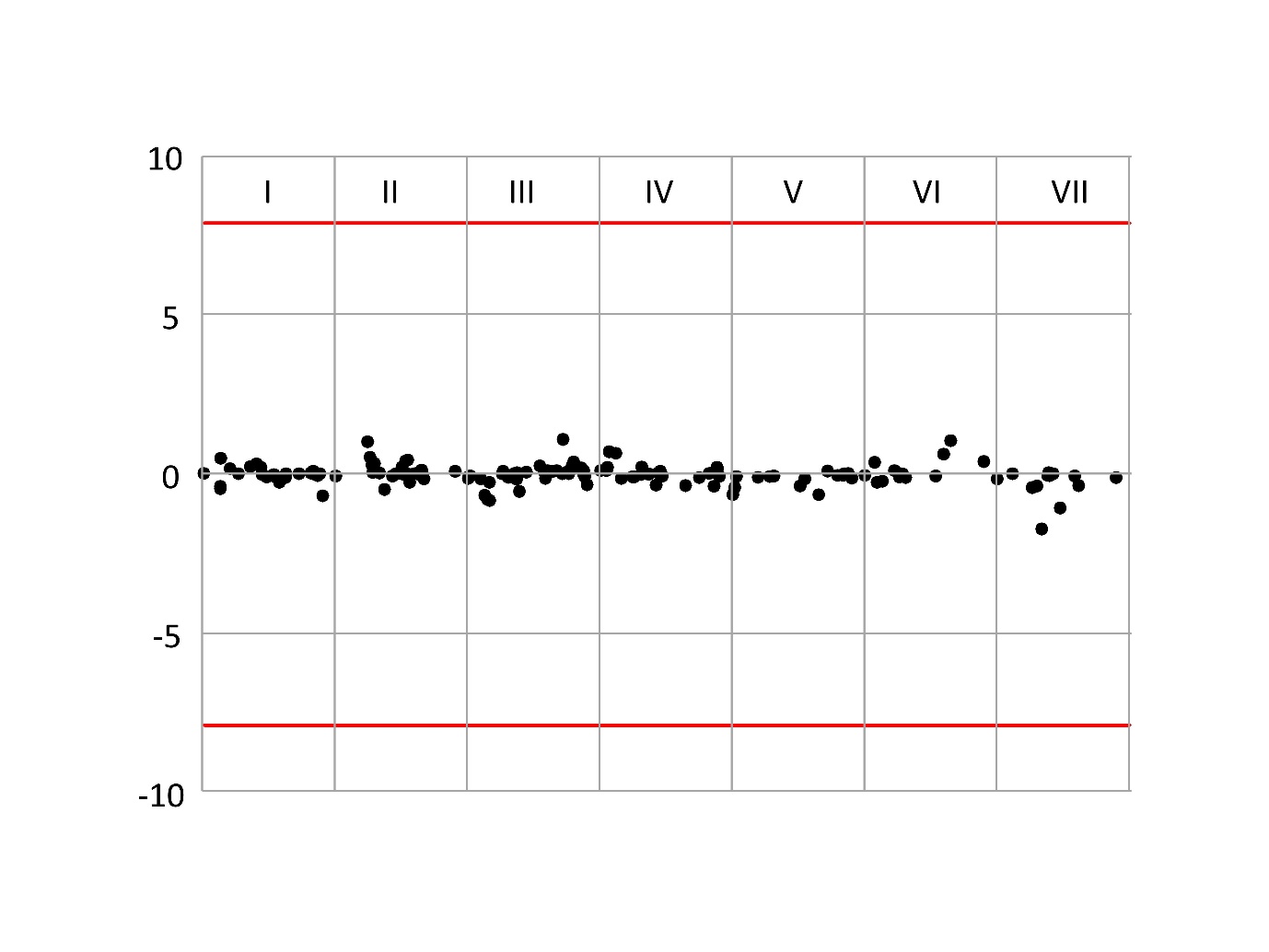


**Figure M2.** Markers are positioned sequentially along the x axis. The x axis value is 0.9 times the percentage distance along the relevant linkage group; successive groups start 100 units apart. The y axis represents the chi square value. The value is positive if the JI 281 or JI 15 allele is more abundant and negative where the JI 399 or JI 1194 allele is more abundant. The red lines correspond to the 0.05% confidence interval.

### *JI 281 x JI 399*

This is the widest of the three crosses (Choi et al., 2004) and for this reason has received most attention. The data set for this map is in the files **Figures M3 and M4.xlsx**. The map is shown in Figure M3.

#### **Figure M3. Linkage map for the JI 281 x JI 399 population**


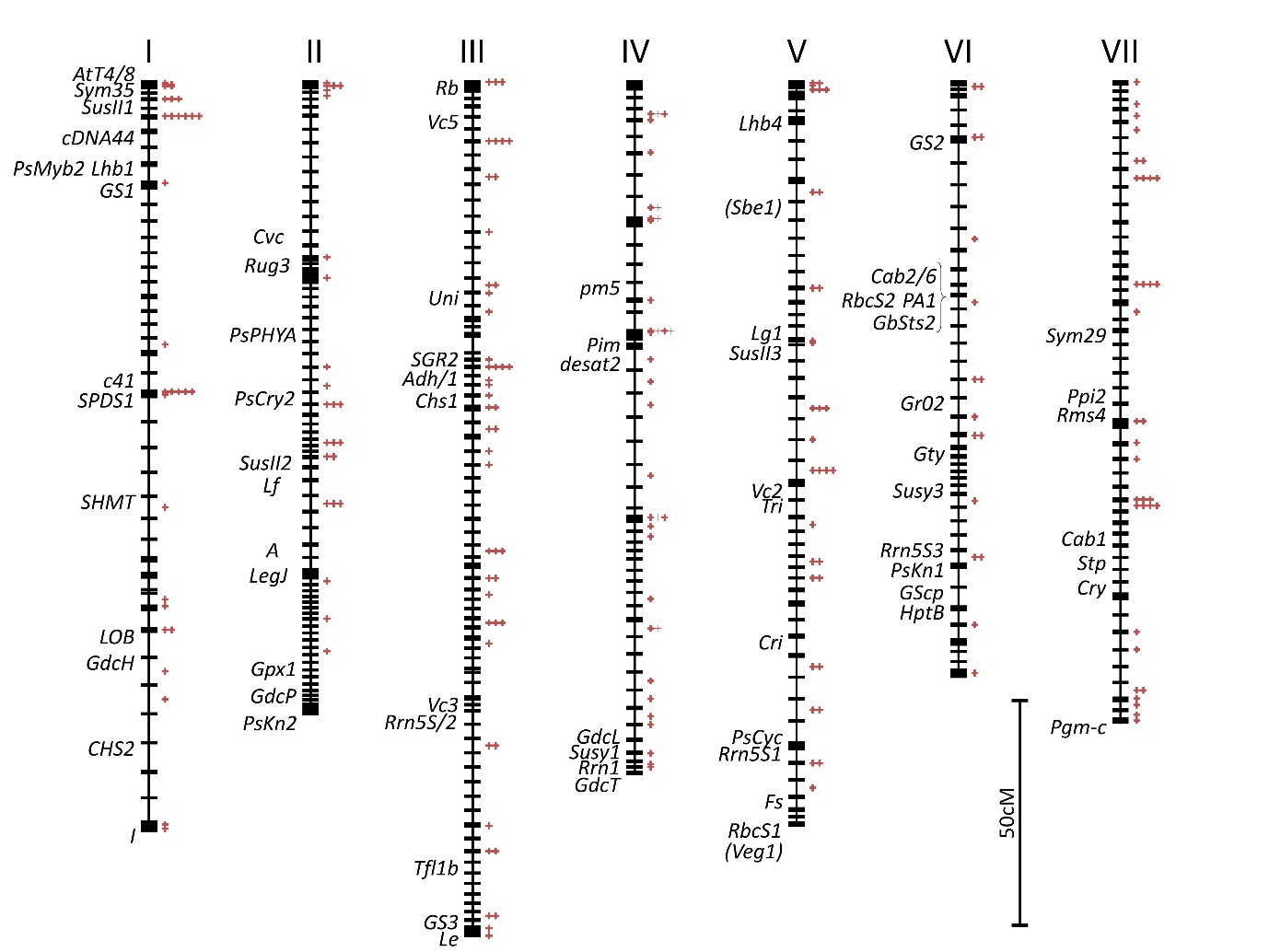


**Figure M3.** The linkage map for JI 281 x JI 399 is illustrated with genetic markers that can be used for orientation or are relevant to the text. The segregation ratios for markers are illustrated in Figure M4. Markers in parentheses are positioned with reference to another marker; for *Veg1* this is the adjacent MADS box gene (Berbel et al. 2012) and for *SbeI* (*R* - Bhattacharyya et al., 1990) this is with reference to adjacent markers that are tightly linked in populations where *R* segregates. Markers indicated with a cross were mapped by Single Strand Conformation Polymorphism (SSCP) in a subset of 16 RILs and positioned either by an exact match of allelic scores or, where a small number of differences were observed by interpolation, according to Kiss et al. (1998). These and other sequence defined markers allow the comparison of this genetic map to sequenced genomes (as shown in Figure M5).

#### **Figure M4. Segregation ratios for the JI 281 x JI 399 population**


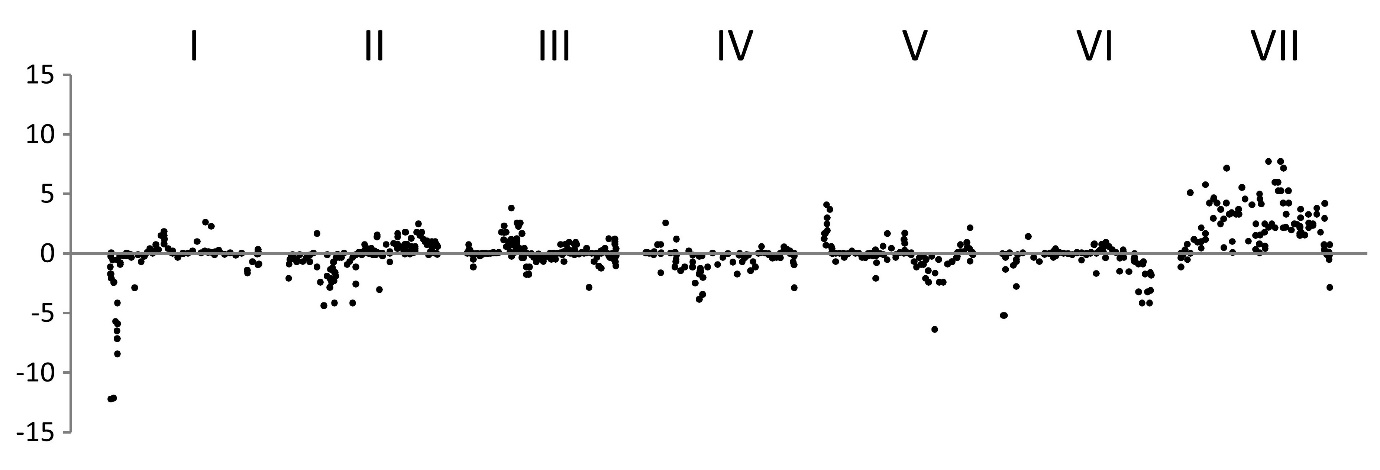


**Figure M4.** Segregation ratios are plotted with the y-axis as χ^2^ values with respect to an equal proportion of each parental allele. Positive values represent an excess of JI 281 alleles and negative values an excess of JI 399 alleles. The x-axis represents the position on the relevant linkage group (indicated as I to VII). The x value represents the map position as a fraction of the length of the linkage group with a short gap between successive linkage groups.

Figure M5 compares the genetic map for JI 281 x JI 399 with the sequence of the *Medicago truncatula* genome v3. The data on which this comparison is made is in **Figure M5.xlsx.**

#### **Figure M5. Comparing the pea genetic map with the M. truncatula genome**


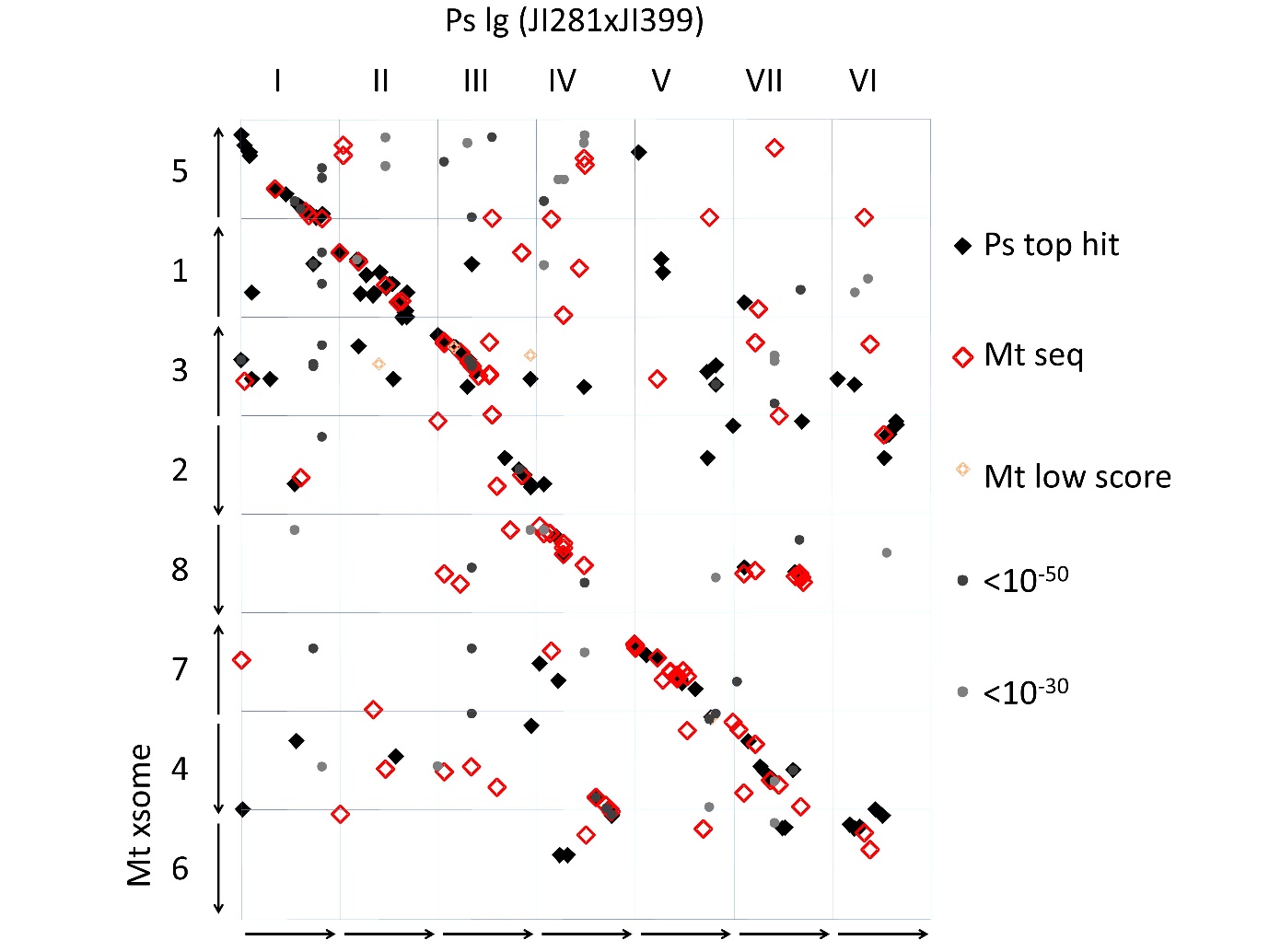


**Figure M5.** Symbols denote degrees of stringency used to identify a BLASTN hit. Symbols marked in red were mapped using primers based on *Medicago truncatula* sequences (and therefore the identity is necessarily 100%). The arrows adjacent to the axes denote the conventional order.

*JI 15 x JI 399*

The JI 15 x JI 399 genetic map is plotted in Figure M6 and the marker segregation ratios shown in Figure M7. The corresponding data set is in the excel files **Figures M6 and M7.xlsx**. Map order was determined by the Threadmapper method (Cheema et al 2010) and map length was scaled with respect to the geodesic distances along the threaded line. Map length was determined from the frequency distribution of recombination events within intervals and the spacing between intervals with recombination events, assuming that a run of exactly x successive non-recombinant intervals occurs at the frequency r^2^n^x^ where r is the frequency of intervals with a recombination event and n (=1-r) is the frequency of non-recombinant intervals. Regression analysis gives an estimate of the total number of recombination events per linkage group from which the recombination rate in a single meiosis and map distance can be calculated according to Haldane and Waddington (1931) and Haldane (1919).

#### **Figure M6. Linkage map** **for the JI 15 x JI 399 population**


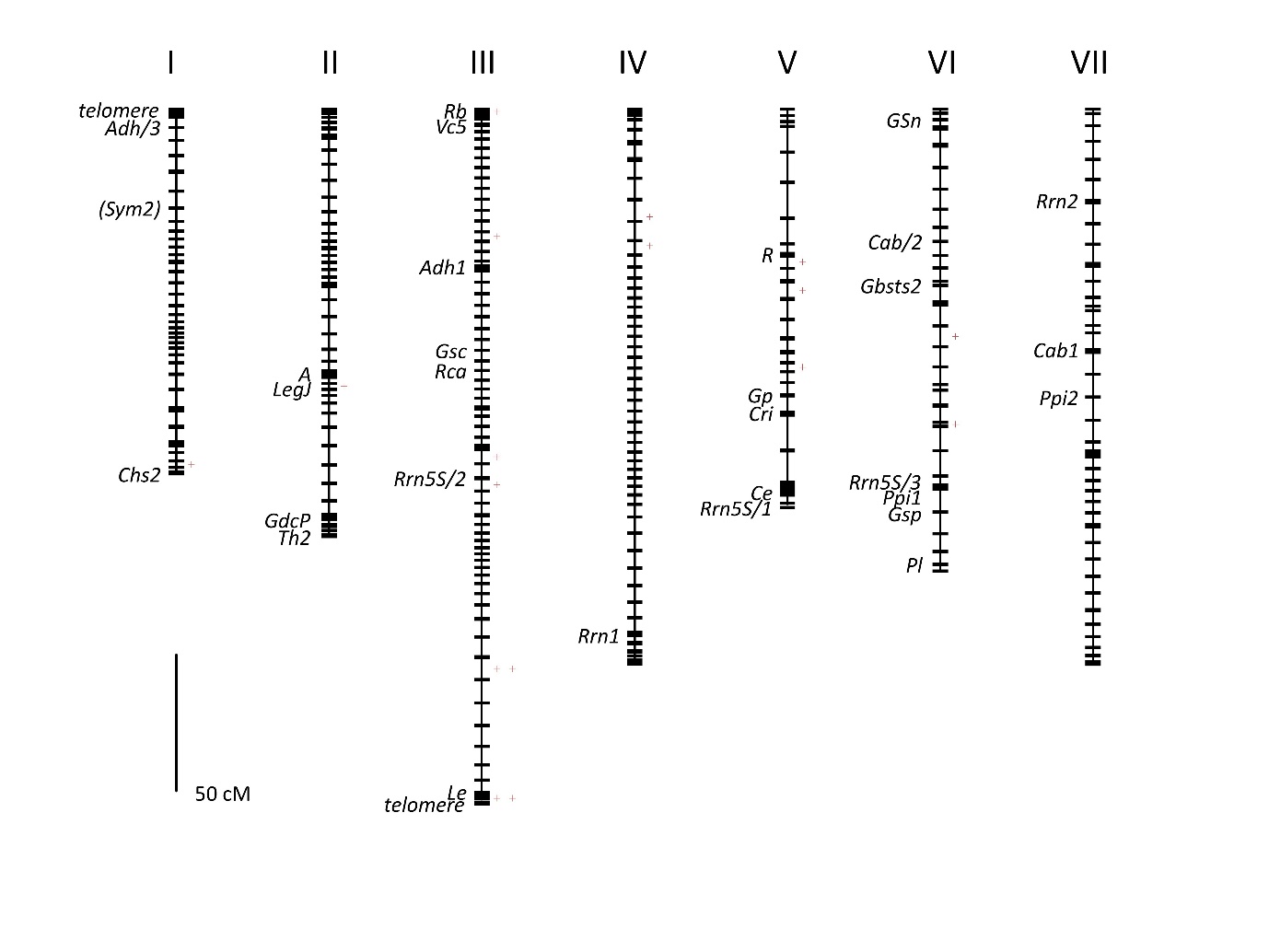


**Figure M6.** The linkage map for JI 15 x JI 399 is illustrated with genetic markers that can be used for orientation or are relevant to the text. The segregation ratios for markers are illustrated in Figure M7. Markers indicated with a cross were mapped by SSCP in a subset of 16 RILs and positioned either by an exact match of allelic scores or, where a small number of differences were observed, by interpolation according to Kiss et al. (1998).

#### **Figure M7. Segregation ratios for the JI 15 x JI 399 population**


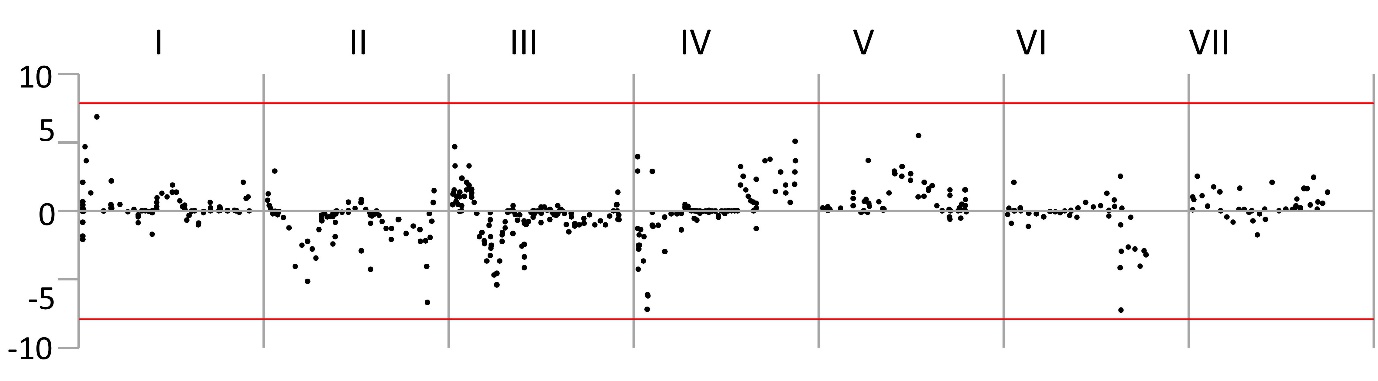


**Figure M7.** Markers are positioned sequentially along the x axis. The x axis value is 0.9 times the percentage distance along the relevant linkage group; successive groups start 100 units apart. The y axis represents the chi-squared value. The value is positive if the JI 15 allele is more abundant and negative where the JI 399 allele is more abundant. The red lines correspond to the 0.05% confidence interval.

*JI 15 x JI 1194*

The JI 15 x JI 1194 genetic map is plotted in Figure M8 and the marker segregation ratios shown in Figure M9. The corresponding data set is in the excel files **Figures M8 and M9.xlsx**. Map order and inter-marker distances were determined by the Threadmapper method (Cheema et al 2010). Map distances correspond to Haldane's mapping function (Haldane 1919).

#### **Figure M8. Linkage map** **for the JI 15 x JI 1194 population**


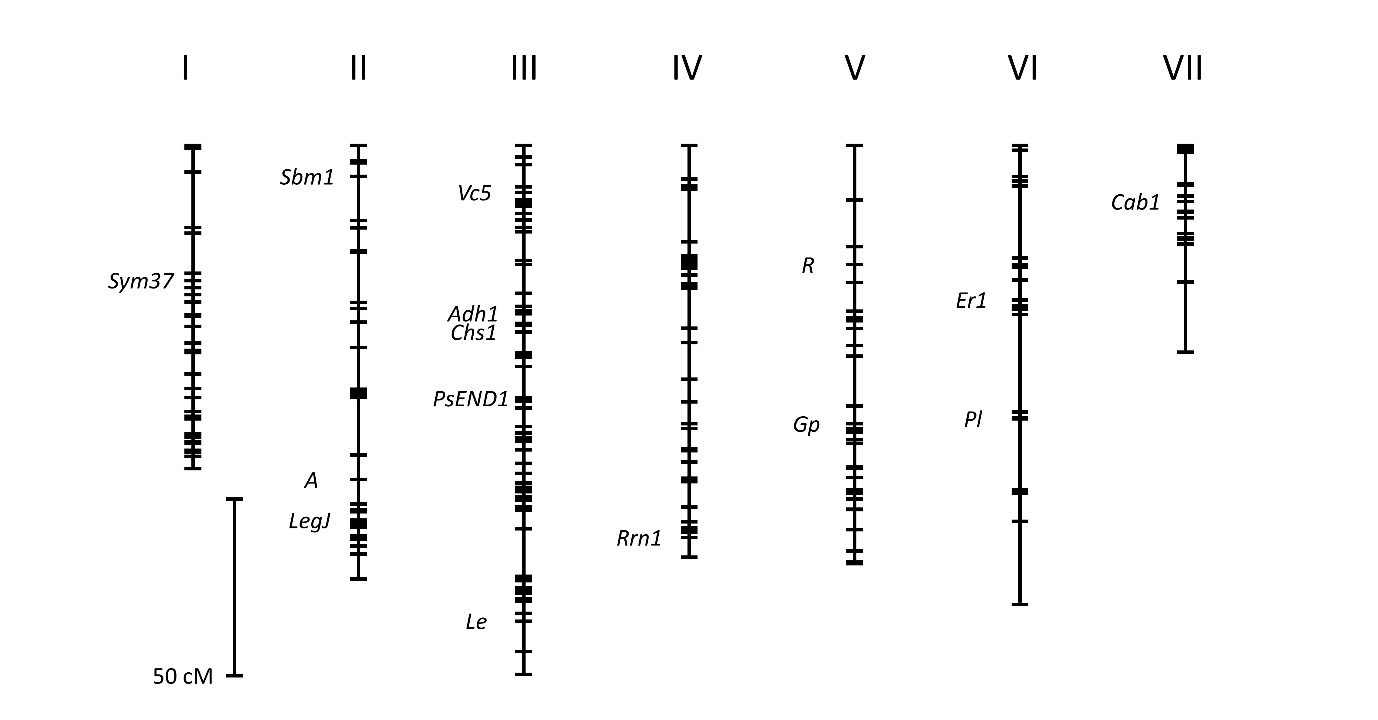


**Figure M8.** The linkage map for JI 15 x JI 1194 is illustrated showing genetic markers that can be used for orientation or are relevant to the text. The segregation ratios for markers are illustrated in Figure M9.

#### **Figure M9. Segregation ratios for the JI 15 x JI 1194 population**


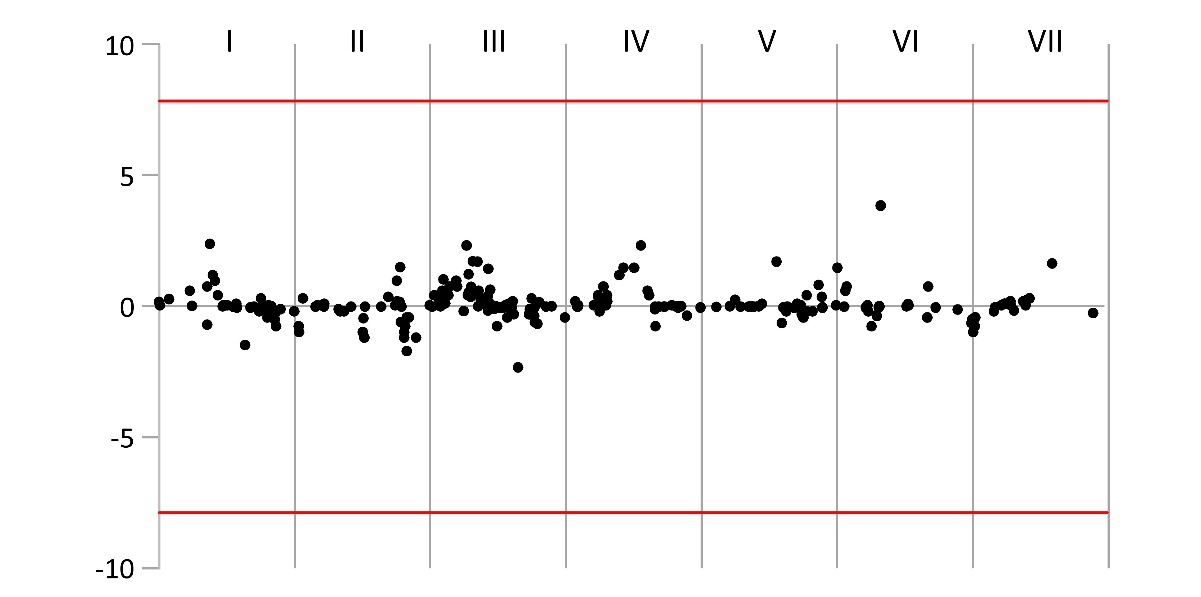


**Figure M9.** Markers are positioned sequentially along the x axis. The x axis value is 0.9 times the percentage distance along the relevant linkage group; successive groups start 100 units apart. The y axis represents the chi-squared value. The value is positive if the JI 15 allele is more abundant and negative where the JI 1194 allele is more abundant. The red lines correspond to the 0.05% confidence interval.

**References**

Berbel, A., Ferrándiz, C., Hecht, V., Dalmais, M., Lund, O. S., Sussmilch, F. C., Taylor, S. A., Bendahmane, A., Ellis, T. H. N., Beltrán, J. P., Weller, J. L and Madueño, F. (2012). *VEGETATIVE1* is essential for development of the compound inflorescence in pea. *Nature Commun.* 3, 797.

Bhattacharyya, M. K., Smith, A. M., Ellis, T. H. N., Hedley, C. and Martin, C. (1990). The wrinkled-seed character of pea described by Mendel is caused by a transposon-like insertion in a gene encoding starch-branching enzyme. *Cell* 60, 115-122.

Cheema, J., Ellis, T. H. N. and Dicks, J. (2010). THREaD Mapper Studio: a novel, visual web server for the estimation of genetic linkage maps. *Nucleic Acids Res.* 38, W188-W193.

Choi, H.-K., Mun, J.-H., Kim, D.-J., Zhu, H., Baek, J.-M., Mudge, J., Roe, B., Ellis N., Doyle, J., Kiss G. B., Young, N. D. and Cook, D. R (2004). Estimating genome conservation between crop and model legume species. *Proc. Natl. Acad. Sci.* USA 101, 15289–15294.

Ellis, T. H. N., Poyser, S. J., Knox, M. R., Vershinin, A. V. and Ambrose M. J. (1998). *Ty1-copia* class retrotransposon insertion site polymorphism for linkage and diversity analysis in pea. *Mol. Gen. Genet.* 260, 9-19.

Ellis, T. H. N. and Poyser, S. J. (2002). An integrated and comparative view of pea genetic and cytogenetic maps. *New Phytol.* 153, 17-25.

Haldane, J. S. B. (1919). The combination of linkage values, and the calculation of distance between loci of linked factors. *Journal of Genet.* 8, 299–309.

Haldane, J. S. B. and Waddington, C. H. (1931). Inbreeding and linkage. *Genet.* 16: 357-374.

Hall, K. J., Parker, J. S. and Ellis T. H. N. (1997a). The relationship between genetic and cytogenetic maps of pea. I. Standard and translocation karyotypes. *Genome* 40, 744-754.

Hall, K. J., Parker, J. S., Ellis, T. H. N., Turner, L., Knox, M. R., Hofer, J. M. I., Lu, J., Ferrandiz, C., Hunter, P. J., Taylor, J. D. and Baird, K. (1997b). The relationship between genetic and cytogenetic maps of pea. II. Physical maps of linkage mapping populations. *Genome* 40, 755-769.

Kalό, P., Seres, A., Taylor, S. A., Jakab, J. and Kevei, Z. (2004). Comparative mapping between *Medicago sativa* and *Pisum sativum*. *Mol. Genet. Genom.* 272, 235–246.

Kiss, G. B., Kereszt, A., Kiss, P. and Endre, G. (1998). Colormapping: a non-mathematical procedure for genetic mapping. *Acta Biol. Hung.* 49, 125-142.

Knox, M., Moreau, C., Lipscombe, J., Baker, D. and Ellis N. (2009). High-throughput retrotransposon-based fluorescent markers: improved information content and allele discrimination. *Plant Meths.* 5, 10.

Lacou, V., Haurogné, K., Ellis N. and Rameau, C. (1998). Genetic mapping in pea. 1- RAPD-based genetic linkage map of *Pisum sativum*. *Theor. Appl. Genet.* 97, 905-915.
